# Gibbs–Helmholtz Graph Neural Network for the Prediction of Activity Coefficients of Polymer Solutions at Infinite Dilution

**DOI:** 10.1021/acs.jpca.3c05892

**Published:** 2023-11-09

**Authors:** Edgar Ivan Sanchez Medina, Sreekanth Kunchapu, Kai Sundmacher

**Affiliations:** † Chair for Process Systems Engineering, Otto-von-Guericke University, Universitätsplatz 2, Magdeburg 39106, Germany; ‡ Process Systems Engineering, Max Planck Institute for Dynamics of Complex Technical Systems, Sandtorstraße 1, Magdeburg 39106, Germany

## Abstract

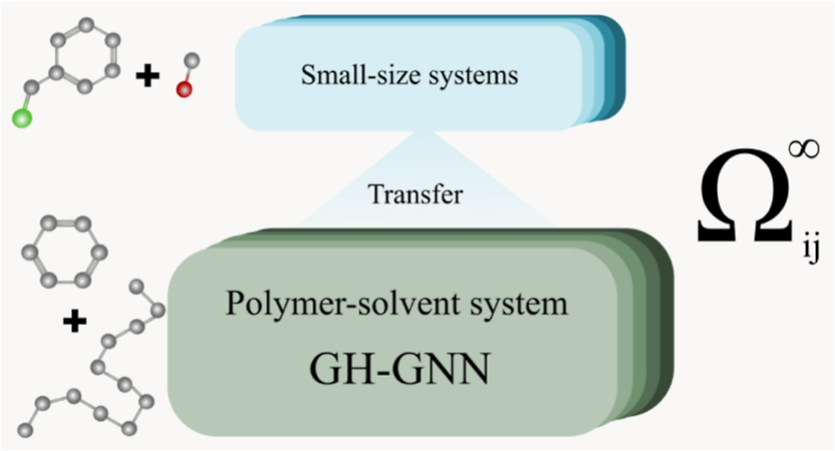

Machine learning models have gained prominence for predicting pure-component properties, yet their application to mixture property prediction remains relatively limited. However, the significance of mixtures in our daily lives is undeniable, particularly in industries such as polymer processing. This study presents a modification of the Gibbs–Helmholtz graph neural network (GH-GNN) model for predicting weight-based activity coefficients at infinite dilution (Ω_*ij*_^∞^) in polymer solutions. We evaluate various polymer representations ranging from monomer, repeating unit, periodic unit, and oligomer and observe that, in data-scarce scenarios of polymer–solvent mixtures, polymer representation specifics have a reduced impact compared to data-rich environments. Leveraging transfer learning, we harness richer activity coefficient data from small-size systems, enhancing model accuracy and reducing prediction variability. The modified GH-GNN model achieves remarkable prediction results in mixture interpolation and solvent extrapolation tasks having an overall mean absolute error of 0.15, showcasing the potential of graph-neural-network-based models for property prediction of polymer solutions. Comparative analysis with the established models UNIFAC-ZM and Entropic-FV suggests a promising avenue for future research on the use of data-driven models for the prediction of the thermodynamic properties of polymer solutions.

## Introduction

Polymer solutions play a vital role in today’s modern lifestyle, with one prominent example being their use in the production of plastics. Plastics have become a ubiquitous material, surpassing the production volume of almost all other material ever produced by humankind.^1^ Modeling the thermodynamic behavior of polymer–solvent systems is essential for the design and optimization of the involved industrial processes.^2−4^ Predictive models are of particular importance given that the experimental determination of all polymer–solvent combinations of interest at different temperatures, pressures, and composition states is an unfeasible task. This becomes even more important in the current transition of industry toward more sustainable pathways. Here, screening large chemical spaces could potentially aid in designing and selecting more sustainable replacements for existing compounds. As shown by a recent survey,^5,6^ the preference for predictive methods is also apparent from an industrial perspective.

Activity coefficients are key for describing the phase equilibria of nonideal solutions such as the ones involving polymers and small-size molecules. They account for deviations from Raoult’s law and are a function of temperature and composition of the mixture and, of course, a function of the chemical species involved. The dependency of activity coefficients on pressure is often of minor importance and hence negligible, especially in low-to medium-pressure regimes.^7^ As the composition of one component in the mixture tends to zero, the prediction of its activity coefficient (referred to as activity coefficient at infinite dilution) becomes harder.^8^ However, estimating activity coefficients at infinite dilution is relevant for their application in environmental studies,^9^ design of separation systems,^10^ and parametrization of models that describe the whole composition range.^11,12^

Existing predictive models for polymer activity coefficients are mostly modifications of the group contribution method UNIFAC^13^ (e.g., UNIFAC-FV,^14^ Entropic-FV,^15^ and UNIFAC-ZM^16^). These methods introduced a free-volume term or a correction in the polymer volume parameter to account for the large differences in molecular sizes that appear in a polymer–solvent system. However, the predictive power of these methods is limited by the feasibility of fragmenting chemical species into UNIFAC functional groups and, in the case of Entropic-FV, by the requirement for accurate knowledge of the molar volumes of the polymer and solvent. Another predictive approach involves using COSMO-based models.^17,18^ However, their reliance on expensive quantum-chemical calculations and conformer searches hinders their practical application for screening large chemical spaces.

When dealing with polymer solutions, molar-based activity coefficients (commonly denoted as γ) lack a clear definition. This ambiguity arises from the fact that a polymer’s molecular mass exists as a distribution rather than as a well-defined value. Additionally, the significant difference in molecular masses between a polymer *j* and a smaller sized solvent molecule *i* complicates the use of the molar fraction as a concentration unit.^14^ For these reasons, Patterson et al.^19^ proposed the use of a weight fraction activity coefficient defined by
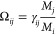
1where *M* refers to the molar mass of the compound. The difficulty of using γ for polymer solutions becomes evident when examining the following expression, which is used to determine activity coefficients at infinite dilution through inverse gas chromatography^19^
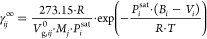
2where *R* denotes the universal gas constant, *V*_g,*ij*_^0^ denotes the specific retention volume corrected to 273.15 K, *P*_*i*_^sat^ denotes the vapor pressure of the solvent, *B*_*i*_ is the second-virial coefficient of the solvent, *V*_*i*_ denotes the molar volume of solvent, and *T* denotes the system’s temperature.

Notice that by introducing eqs 1 into 2, the resulting expression becomes independent from the polymer’s molar mass *M*_*j*_ and the activity coefficients at infinite dilution of a polymer solution can be directly measured from the retention volume
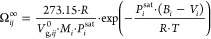
3

As pointed out by a recent survey, there is still a need for further development of predictive polymer solution models.^6^ This paper aims to broaden the research in this direction by extending a recently proposed model based on graph neural networks (GNN) [referred to as the Gibbs–Helmholtz graph neural network (GH-GNN)^20^] to predict infinite dilution activity coefficients of polymer solutions, and by providing a curated and readily accessible data set for benchmarking models developed for this purpose. The latter addition is particularly valuable, considering the significant lack of well-curated data sets in polymer informatics.^21−23^

In the last couple of years, there has been growing interest in using GNNs for polymer property prediction.^24−31^ However, all of these works have focused on predicting properties of pure polymers. To the best of our knowledge, no previous attempts have been made to study the performance of GNNs for predicting properties of polymer solutions in general, and Ω_*ij*_^∞^, for polymer solutions in particular.

This article is structured as follows: in the first section, we present the modifications made to the GH-GNN model to compute activity coefficients at an infinite dilution of polymer solutions. We also discuss the different polymer representations studied, including (i) monomer, (ii) repeating unit, (iii) periodic unit, and (iv) oligomer. Additionally, we explain the transfer learning approach from small-sized systems to polymer solutions. In the second section, we provide a detailed description of the data set, the cleaning process, and the splitting methods used to train and assess the models. In the third section, we present the results for the predictions made using different polymer representations, the impact of transfer learning, and the performance of the modified GH-GNN model for interpolating different mixtures and extrapolating them to unobserved solvents. Furthermore, we compare the modified GH-GNN model to the Entropic-FV and UNIFAC-ZM models in terms of the prediction accuracy. Finally, in the last section, we present the conclusions of this work along with some recommendations for future research.

## Methods

### Modification of the GH-GNN

The original GH-GNN model^20^ uses (molecular) graphs as defined by Battaglia et al.^32^ containing node-, edge-, and global-level features. In these vectorial features, information about the specific molecule is stored regarding the atoms (node-level information), the chemical bonds (edge-level information), and the polarity and polarizability of the molecule (global-level information). The solute and solvent graphs are then passed through a molecular-level GNN and pooled to vector embeddings that are used to create another graph that represents the mixture. In this mixture graph, each node represents a chemical species, and edges represent hydrogen bonding interactions. This mixture graph is later passed through a second (mixture-level) GNN and then pooled to a vector termed the mixture fingerprint (MF). Finally, this fingerprint is utilized to estimate the temperature-independent parameters of an expression derived from the Gibbs–Helmholtz equation, which predicts the activity coefficient at infinite dilution. We refer the reader to our previous work^20^ for further details on the model.

**Figure 1:**
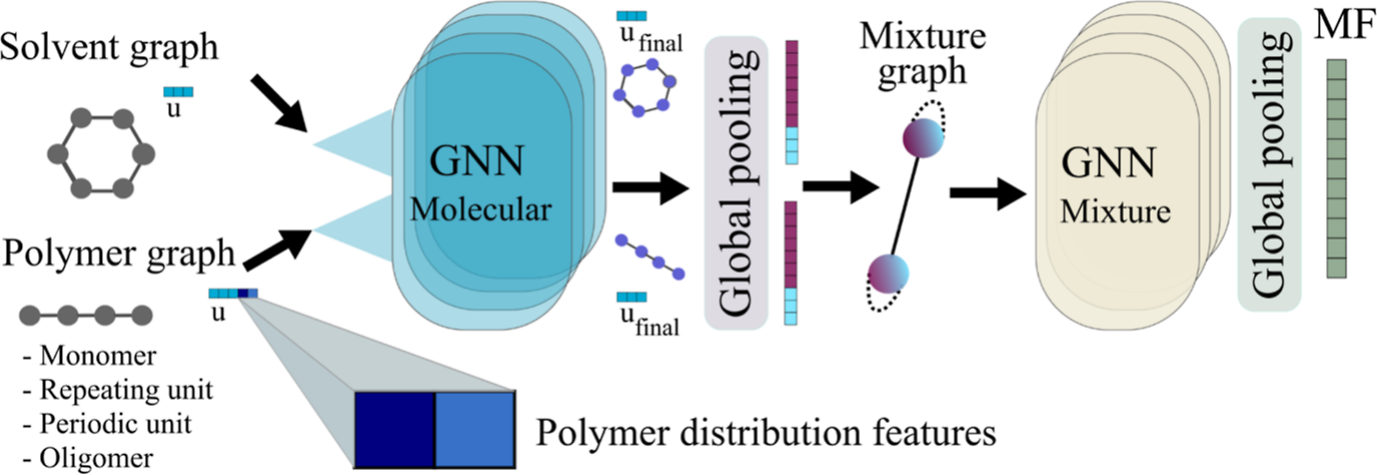
Modifications applied to the GH-GNN model^20^ to predict polymer solutions. In this context, the solute is a polymer, and its representation can vary, including the corresponding monomer(s), repeating unit, periodic unit, or oligomer. The polymer’s molar mass distribution information [ln(*M*_n_) and/or ln(*M*_w_)] is integrated into the polymer graph’s global-level features via concatenation. The solvent and polymer graphs undergo distinct first message passing layers, while a shared second message passing layer processes both solvent and polymer graphs. Subsequently, the mixture graph is constructed and processed through a mixture-level GNN, yielding the MF. This fingerprint is then utilized to regress the parameters  and *K*_2,*ij*_ in the Gibbs–Helmholtz-derived expression (eq 4).

In this work, we have used the same graph definition from Battaglia et al.^32^ and the same node- and edge-level features as in our previous work.^20^ Similarly, we constructed one graph for the solvent and one graph for the solute (now a polymer). For our study, the first set of questions emerges: how can we effectively represent a polymer as a molecular graph given its inherent polydispersity? What is the most beneficial polymer representation? As mentioned above, a polymer cannot be defined as a fixed molecular structure. Instead, it is usually characterized with a measure of its polydispersity (e.g., via the number-average molar mass *M*_n_ or the weight-average molar mass *M*_w_). For this reason, we have now included molecular mass distribution information as part of the solute’s global-level feature vector, as illustrated in Figure 1. The natural logarithm [i.e., ln(*M*_n_) and/or ln(*M*_w_)] is used here for scaling purposes, given its ability to normalize a wide range of values. It is worth noticing that in most experimental papers reporting infinite dilution activity coefficients of polymer solutions, only *M*_n_ and/or *M*_w_ is reported, while in others, none of them are mentioned. Information about the tacticity of the polymer samples is even rarer and, therefore, not included here as part of the modeling framework. However, as more information regarding the polymer tacticity (i.e., the spacial arrangement of regular units along the polymer chain) and the corresponding activity coefficients at infinite dilution become available, it might be relevant for predicting activity coefficients of polymer solutions more accurately.

The polymer graph is constructed using one of the possible representations (i.e., either the monomer(s), the repeating unit, the periodic unit, or the oligomer). Further details on the different polymer representations used here are given in the next section. Once the initial solvent and polymer graphs are constructed, they are passed through a molecular-level GNN. In contrast to the original GH-GNN model,^20^ the approach proposed here employs two distinct initial message passing layers within the molecular-level GNN. These layers transform the solvent and polymer graphs separately. This separation accounts for the differing initial dimensions of the corresponding global-level embeddings. However, the second message passing layer is unique despite processing the solvent or polymer graph (i.e., the same model parameters are used for processing the solvent and polymer graphs). While we have introduced this modification in the first message passing layer of the molecular-level GNN, we have retained the same architecture and hyperparameters as the original GH-GNN model.^20^ After the second global pooling, the MF (MF in Figure 1) is generated. This fingerprint serves as a vectorial representation characterizing the specific polymer–solvent mixture. This fingerprint is used to regress the activity coefficient using two independent multilayer perceptrons for predicting  and *K*_2,*ij*_ in eq 4. Hence, the temperature dependency is introduced similarly as in the original GH-GNN model^20^ using eq 4 that results from integrating the Gibbs–Helmholtz equation assuming a temperature-independent partial molar excess enthalpy at infinite dilution *h*_*ij*_^*E*,∞^
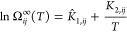
4where  and *K*_2,*ij*_ are temperature-independent parameters for the specific solvent *i* and polymer *j*. The parameter *K*_2,*ij*_ in eq 4 holds the same value in both the original^20^ and modified GH-GNN models, specifically equating to the ratio *K*_2,*ij*_ = *h*_*ij*_^*E*,∞^/*R*. In contrast, the parameter  in eq 4 now takes on the role of the logarithmic weight-based activity coefficient at infinite dilution, as the temperature of the system approaches infinity. Consequently, the corresponding parameters of the original^20^ and modified GH-GNN models are interconnected through the relationship .

### Polymer Representations

The first challenge in polymer informatics is to generate machine-readable representations of the polymer itself.^21,22^ This challenge becomes particularly important when employing machine learning techniques for predicting polymer properties.^33^ In contrast to small molecules, polymers lack well-defined structures. In the literature, three main polymer representations stand-out: the monomer(s) which is a small-size molecule from which the polymer is synthesized, the repeating unit which refers to the section of the polymer chain that is repeated periodically depending on the degree of polymerization, and an oligomer of fixed size representing certain part of the polymer chain. These representations have been systematically studied and compared for predicting the polymer’s glass-transition temperature.^33^ Of these three representations, the monomer has historically been more commonly utilized in the context of polymer solutions. This becomes evident in the early works on UNIFAC-based group contribution methods,^14−16^ and it is still used in other polymer-property prediction tasks.^30^ However, the monomer representation lacks essential information about polymer bonding and periodicity. As a result, there has been a growing shift toward utilizing repeating units or oligomers as more comprehensive representations for predicting polymer properties.^17,18,23,25,27,29^

In the context of GNNs, translating the chosen polymer representative molecule (e.g., monomer, repeating unit, or oligomer) into a molecular graph is crucial. Certain polymer representations offer distinct advantages over others. For instance, a single monomer can result in multiple polymers depending on the polymerization process, as discussed elsewhere.^33^ This becomes evident in cases such as the buta-1,3-diene monomer, which can polymerize into either polyethylene or poly(but-1-ene). As a consequence, the monomer representation may not capture the uniqueness of a polymer. Similarly, the repeating unit representation might also fall short in capturing a polymer uniquely. This concern is exemplified by instances such as poly(vinyl alcohol) and polyethylene glycol, both of which yield the same graph for their repeating units, as discussed elsewhere.^27^

**Figure 2:**
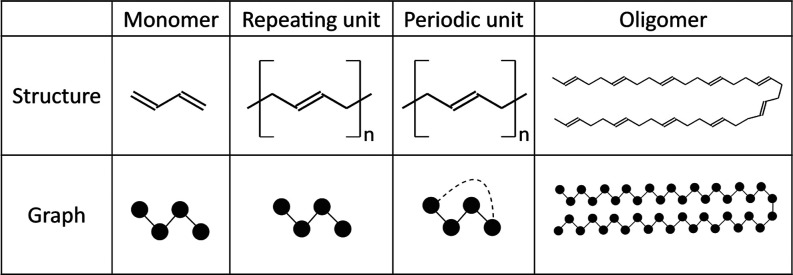
Polymer representations used in this work exemplified by polybutadiene: monomer, repeating unit, periodic unit, and oligomer of size 10. Notice that the edge information between the graph of the monomer and the graph of the repeating unit will be different according to the different distribution of single and double bonds.

In response to these challenges, recent advances have introduced alternative polymer graph representations. One such approach, termed the periodic polymer graph,^27^ extends the traditional repeating unit representation. It involves adding an additional edge that connects the two polymerization points within the repeating unit. This enhancement ensures a unique representation of polymers, encapsulating both bonding and periodicity information. Antoniuk et al.^27^ have reported an overall increase in accuracy when periodic graphs are used compared to the repeating unit graphs. Similarly, an analogous concept has been explored in a separate study,^26^ where molecular graphs representing polymers were enriched by incorporating weighted edges connecting polymerization sites. These edges are assigned weights based on the probability of occurrence within the polymer chain. This augmentation offers an enhanced representation not only for homopolymers but also for copolymers with varying repeating unit compositions. For the scope of this work, we have concentrated on homopolymers, which consist of polymer chains composed of a single repeating unit. As a result, we examined the performance of the periodic unit representation alongside the common monomer, repeating unit, and oligomer representations (see Figure 2).

**Figure 3:**
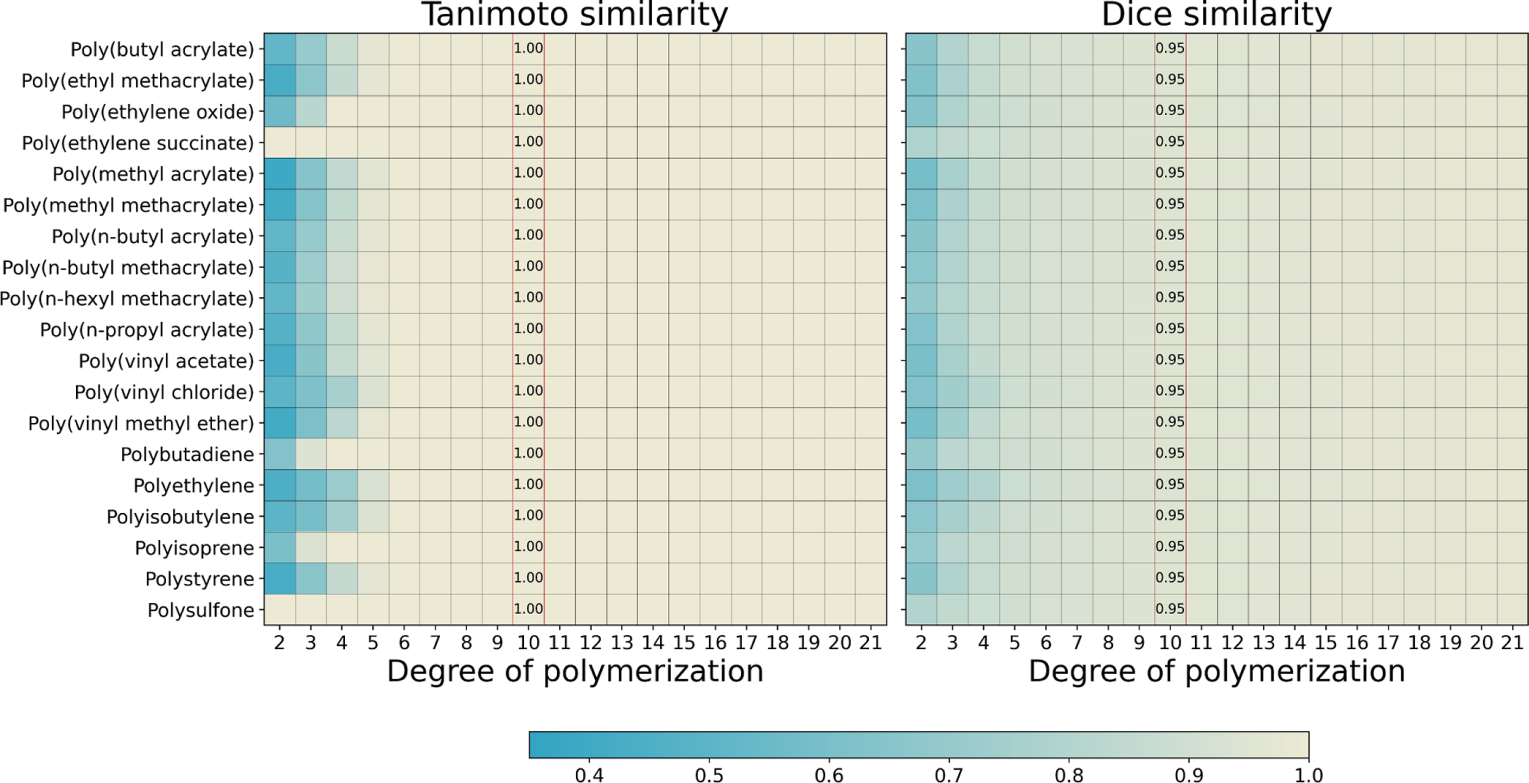
Heatmaps displaying the convergence similarity of oligomers with varying degrees of polymerization. The color of the heatmap indicates the similarity score, with a value of 1 representing the highest similarity. Tanimoto similarity scores were calculated using Morgan binary fingerprints of radius 4 and size 2048, while Dice similarity scores were computed using Morgan count-based fingerprints of radius 4. The degree of polymerization of 10 is highlighted as this is the chosen size for the oligomer representations in this study.

When the oligomer representation is utilized, a critical question emerges: which polymerization degree should be chosen? While previous studies have examined oligomers of sizes two or three,^17^ recent research^33^ highlighted the convergence behavior of molecular fingerprints from synthetic polymers. This is shown in Figure 3 by using the Tanimoto and Dice similarities for some random polymers considered in this work.

In these plots, the fingerprint similarity between oligomers of contiguous sizes is calculated (e.g., similarity between the oligomers of sizes 2 and 3, 3 and 4, 4 and 5, and so on). Tao et al.^33^ decided to use oligomers of size 16 as a trade-off between similarity convergence and computational efficiency. However, as can be observed in Figure 3, the Tanimoto similarity of the oligomers converges to one already at size 6. In comparison, the Dice similarity converges to one at a slower rate.

In this work, we chose an oligomer of size 10 to ensure that the Tanimoto similarity of all polymers considered has converged to one and that the Dice similarity has reached a value of 0.95. For calculating the Tanimoto similarity, Morgan binary fingerprints were used with a radius of 4 and a bit-size of 2048. In the case of the Dice similarity, the count-based Morgan fingerprint was used with a radius of 4. These calculations were carried-out using RDKit.^34^

A random forest model trained on the Morgan binary fingerprints was used as a baseline for comparison to the modified GH-GNN model studied in this work. Regarding the random forest model, the polymer and solvent fingerprints were concatenated along with the temperature and polymer distribution information to obtain the vectorial representation of the specific mixture at the given conditions.

### Transfer Learning

Transfer learning is a valuable technique that involves two key stages: pretraining on a distinct data set, followed by fine-tuning using the target data set. This approach leverages the benefits of the initial training on a larger and contextually relevant data set to enhance the model’s performance on the specific task of interest. Through this process, the model’s parameters are better initialized, enabling it to transfer valuable insights gained from the first task to the second. As a result, the accuracy of the model and applicability for the specific prediction task are substantially improved. This transfer of generalities allows the model to capture underlying patterns and relationships present in the initial data set, which can then be adapted to enhance its predictive capabilities for the target data set. Several successful applications of transfer learning for thermodynamic property prediction of solutions have been reported in the literature.^20,35,36^

In this study, we explore the impact of transfer learning by initially pretraining the modified GH-GNN model on the DECHEMA data set, which was also utilized in our previous publication.^20^ This data set comprises 40,219 experimental activity coefficient values at infinite dilution for mixtures involving small-size molecules. To ensure consistent pretraining, we first calculated the weight-based activity coefficients at infinite dilution, denoted as Ω_*ij*_^∞^, from the original γ_*ij*_^∞^ values using eq 1. The same data splitting (based on chemical classes) reported in the original publication^20^ was used here for pretraining the model and validating its pretrained performance. The model was pretrained during 200 epochs until the validation loss converged. The pretraining convergence plots are available in the Supporting Information. After the pretraining, we fine-tuned the model on the polymer data set of this work.

## Data Sets

### Data Source

The data set employed in this study comprises Ω_*ij*_^∞^ values for binary mixtures of polymers and small-size solvents. These values were derived from inverse gas chromatography measurements reported in the scientific literature. The data set was originally collected in volume XIV of the DECHEMA Chemistry Data Series.^37^ For this study, our analysis centered exclusively on homopolymers, which constitute the majority of the data available in the aforementioned collection. We have corrected several errors present in the original data collection (see Supporting Information for a detailed description of the corrections) and have carefully collected SMILES strings for all solvents and polymers present in the data set. This includes the SMILES representation of the monomer(s), repeating units, periodic units, and repeating units with polymerization points from which the oligomers’ SMILES can be generated for different degrees of polymerization. The SMILES containing the polymerization points uses a “*” for indicating the connection points. For example, the SMILES “C*C=CC*” represents the depicted repeating unit of polybutadiene in Figure 2. The data points that do not report any information about the polymer molar mass distribution of the samples used were discarded. Repeated measurements were averaged to obtain a single point to account for the uncertainty in the data. However, similar to the case of IDACs for small-size systems, a rigorous estimation of the experimental data uncertainty is difficult due to the lack of reported compound purities and standard deviation of replicate measurements in most of the literature (e.g., in volume XIV of the DECHEMA Chemistry Data Series^37^). However, some uncertainty estimations of weight-based IDACs can be found in the literature. Belusso et al.^38^ has reported uncertainties to the reported measurements properly. They reported an averaged relative uncertainty of Ω^∞^ of 9.5%. Similarly, Sadowski et al.^39^ and Domańska and Żołek-Tryznowska^40^ have reported an uncertainty of around 5%, and Wang et al.,^41^ an average uncertainty of 3.7%. Uncertainty estimation of ln Ω^∞^ has been reported as 2.5% by Sørensen et al.^42^

The curated data set consists of 48 distinct homopolymers and 150 solvents. Some of the measurements report number-average molecular mass *M*_n_, some other report the weight-average molecular mass *M*_w_, and some report both. We have then created three distinct data sets (*M*_n_ data set, *M*_w_ data set and *M*_n_/*M*_w_ data set) to study the influence of the different molar mass distribution descriptors on the prediction of Ω_*ij*_^∞^. The number of points, distinct polymers, and distinct solvents on each data set is shown in Table 1. The percentage of polymer–solvent observations in each data set compared to all possible combinations of the corresponding polymers and solvents is also indicated. It is important to mention that for all three data sets, over 77% of the observations are associated with the top 8 most popular polymers within each data set. Similarly, approximately 50% of the observations correspond to the top 15 most popular solvents (Supporting Information).

**Table 1:** Information on the Three Distinct Data Sets Used in This Work^a^

	data set		
information	*M*_n_	*M*_w_	*M*_n_/*M*_w_
no. points	2532	2763	1666
polymers	42	28	22
solvents	137	122	107
% observed	10.71	16.04	16.19

a *M*_n_ refers to the number average molecular mass and *M*_w_ refers to the weight average molecular mass. The percentage of polymer–solvent observations compared with all possible combinations of the given polymers and solvents is also indicated.

The data sets primarily comprise synthetic thermoplastics, but some rubbers and resins are also included. The complete list of polymers is available in the GitHub repository. The data distribution of the covered temperatures, and *M*_n_ and *M*_w_ values for each data set is available in the Supporting Information. It is important to note that due to the challenge of obtaining uncertainty estimations for each data point in the database, we have not explicitly incorporated experimental uncertainty into this work, apart from the previously mentioned averaging of repeated measurements. However, if uncertainty estimations for individual data points become accessible, then they could be leveraged to assign weights to each data point in the loss function during training, reflecting their respective uncertainty levels.

### Data Splitting

Each of the three data sets was split to evaluate the models’ performance in two tasks: interpolation among mixtures and extrapolation to new solvents. In the interpolation task, the model is trained on a subset of polymer–solvent mixtures and tested on distinct polymer–solvent combinations, where the individual polymers and solvents are seen during training but in different combinations. On the other hand, in the extrapolation task, the model is tested on polymer–solvent combinations that include solvents not encountered during training, allowing an assessment of its ability to handle unseen solvents.

For testing interpolation, first, the random selection of a subset of polymer–solvent mixtures (90% of all unique combinations) is selected to form the training set. The remaining mixtures formed the test set. To ensure the test set only contains interpolation cases, any mixture containing an unseen polymer or solvent is reassigned to the training set. To ensure a robust estimation of the models’ performance, 10 independent splits are performed using different random seeds, and they are later used to train/test the models. The reported performance metrics correspond to the average performance across the 10 splits, unless stated otherwise. The proportion of training points was kept on average as 90.5% for all three data sets (i.e., for the *M*_n_, *M*_w_ and *M*_n_/*M*_w_ data sets).

For testing extrapolation, first a list of unique solvents is formed. Then, a random selection of (90%) of the unique solvents is carried out. The training set is composed of all polymer–solvent mixtures containing any of these solvents, while the testing set comprises the remaining unique mixtures not included in the training set. This ensures that only polymer–solvent combinations in which the solvent has not been seen during training are included in the test set, enabling the evaluation of extrapolation performance. Similar to the interpolation case, the models are evaluated using 10 independent splits with different random seeds, and the reported metrics are the averages across these 10 splits. The average proportion of training points for all three data sets (i.e., *M*_n_, *M*_w_, and *M*_n_/*M*_w_) was on average 88.9%.

## Results and Discussion

### Polymer Representations

**Figure 4:**
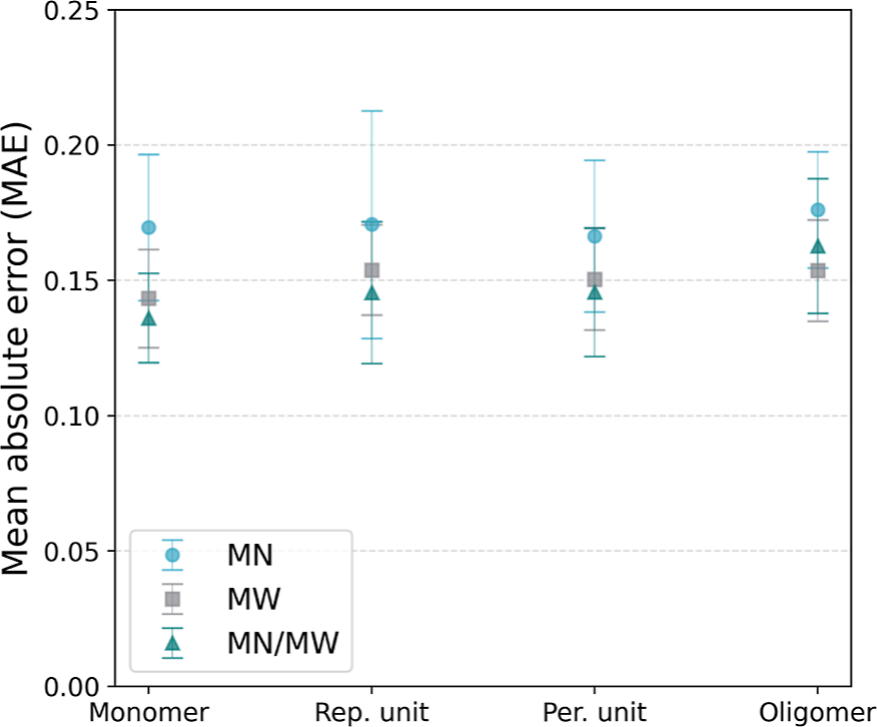
Performance of the modified GH-GNN model (without pretraining) according to the MAE on the test set for the interpolation task. The markers and the error bars show the average and standard deviation, respectively, across the 10 splits. The results are shown for the 3 different data sets (i.e., *M*_n_, *M*_w_, and *M*_n_/*M*_w_) using the four different polymer representations.

To determine the preferable polymer representation for enhancing model accuracy, we assessed the performance of the modified GH-GNN model (without pretraining) through the interpolation task. The results are depicted in Figure 4, showcasing the mean absolute error (MAE) achieved for the test set. Each marker represents the average, while the error bars correspond to the standard deviation, computed across the 10 different splits of the data. These outcomes are presented for the three distinct data sets. On two (*M*_w_ and *M*_n_/*M*_w_) out of the three data sets, the monomer representation shows the lowest MAE on average. On the *M*_n_ data set, the periodic unit representation achieved on average the lowest MAE.

However, we could not observe a statistically significant disparity among the polymer representations for the Ω_*ij*_^∞^ case studied here based on model’s accuracy. This is shown by the overlapping error bars across all polymer representations for each data set. This contrasts with the findings of Antoniuk et al.,^27^ who noted an overall superior performance with the periodic unit in comparison to the repeating unit. However, several significant distinctions should be acknowledged: first, the study by Antoniuk et al.^27^ solely addressed property predictions for pure polymers, whereas the Ω_*ij*_^∞^ property explored here is dependent upon both the solvent and the polymer. This can reduce the overall influence of the polymer representation on the final prediction. Second, the outcomes presented by Antoniuk et al.^27^ were derived from a single, randomly selected test set. In contrast, we considered ten different randomly selected test sets, leading to the availability of error bars in our case that can help assess the statistical significance of the different representations. Third, and perhaps the most important, while Antoniuk et al.^27^ constructed their models using 9935 distinct homopolymers, our study encompasses only 48 homopolymers. This suggests that in low-data regimes, the precise polymer representation might not be as significant for the model accuracy as in higher data regimes. This is of course subject to the default drawbacks of some polymer representations, as explained already in the Methods section. It also highlights the scarcity of polymer-solution data compared with pure polymer data. The same lack of statistical significance for the performance of different polymer representations is observed in the case of the baseline random forest model trained on Morgan binary fingerprints and the pretrained and fine-tuned GH-GNN model in both interpolation and extrapolation tasks (see Supporting Information).

The limited number of points and polymers in the present data set makes it difficult to elucidate the default drawbacks of the monomer and repeating unit representations that are explained in the Methods section. Regarding the main drawback of the monomer representation (i.e., a single monomer representing different polymers), the data set only contains the case of polyethylene and polyethylene low-density polymers sharing the same monomer structure. While these two polymers indeed share the same basic chemical structure, their molecular arrangements differ from each other due to different degrees of branching. Capturing these differences remains a challenge for the polymer representations studied in this work. Similarly, the main drawback of the repeating unit (i.e., single repeating unit representing different polymers) cannot be observed, as all polymers considered here have different repeating unit representations. Based on the theoretical advantages and the similarity in the model’s accuracy, we have chosen the periodic unit representation in the following analyses.

Furthermore, by looking at the error bars in Figure 4, it is noteworthy that the performance variability of the modified GH-GNN is greater for the *M*_n_ data set compared to the remaining two. This particular data set covers a broader binary chemical space compared with the others. Specifically, it includes approximately 35 and 10–15% more polymers and solvents, respectively. The same trend in performance variability can be observed in the case of the random forest model and the pretrained and fine-tuned GH-GNN model (see Supporting Information). On average, the model trained with both *M*_n_ and *M*_w_ achieves lower errors compared to the models trained using only the individual polymer distribution information. However, the difference in performance is within the error bars. Hence, no significant difference can be observed between the models using these different characterizations of the polymer distributions.

### Transfer Learning

**Figure 5:**
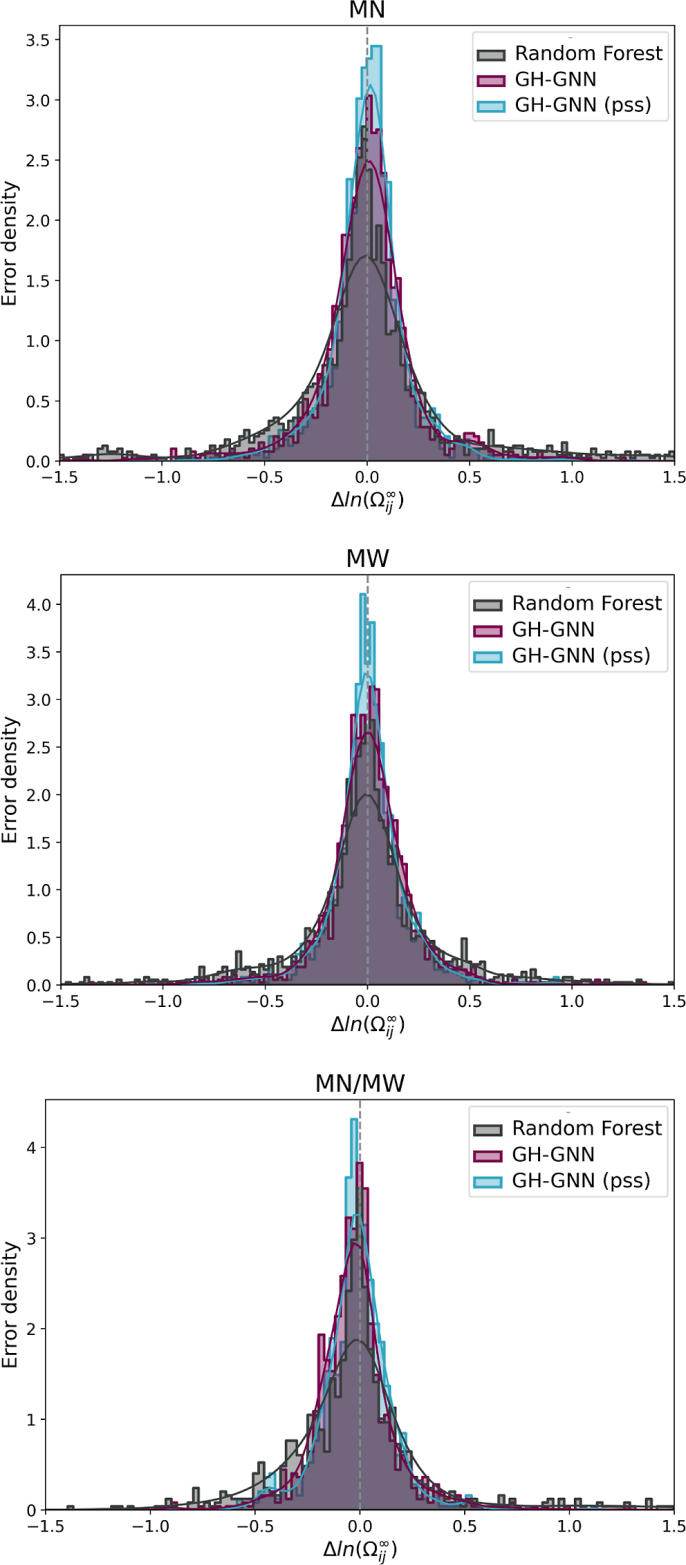
Absolute error density obtained from the random forest, the modified GH-GNN (GH-GNN) and the pretrained and fine-tuned modified GH-GNN [GH-GNN (pss)] models trained using the periodic unit. The results are shown for the interpolation task and are averaged across the 10 splits by using the test set.

Incorporating transfer learning significantly enhanced the prediction accuracy of the modified GH-GNN model. To visually present this improvement, Figure 5 displays the absolute error density achieved by the modified GH-GNN models. This figure compares the performance between the model with pretraining (indicated as “pss” for pretrained in small systems) and the model without pretraining, both utilizing the periodic unit representation for the interpolation task. Additionally, the performance of the random forest baseline is provided for reference.

Across all data sets, the GNN-based models consistently outperform the random forest baseline. This observation underscores the efficacy of graph molecular and mixture representations in capturing data patterns, leading to more accurate predictions compared with traditional molecular fingerprints. Incorporating transfer learning resulted in a reduction of the averaged MAE for the modified GH-GNN model, by 23.5, 13.3, and 13.3% for the *M*_n_, *M*_w_, and *M*_n_/*M*_w_ data sets, respectively (cf. Table 2). This reduction highlights the effectiveness of transfer learning as a versatile tool applicable to diverse scenarios characterized by data scarcity, specifically in the prediction of the activity coefficients of polymer solutions. In addition to the improvements in accuracy, the introduction of transfer learning led to a significant reduction in prediction variability within the GH-GNN model. This reduction becomes evident when contrasting the standard deviation values between the GH-GNN (pss) model and the GH-GNN model trained directly on the data, as presented in Table 2. This observation underscores that transfer learning not only enhances model accuracy but also improves the consistency and, therefore, reliability of the predictions. This is a paramount point when designing models intended for industrial applications.

**Table 2:** MAE and Coefficient of Determination (*R*^2^) Achieved by the Models During Interpolation and Extrapolation^a^

	interpolation			extrapolation		
MAE ↓	*M*_n_	*M*_w_	*M*_n_/*M*_w_	*M*_n_	*M*_w_	*M*_n_/*M*_w_
random forest	0.27 (0.07)	0.22 (0.03)	0.27 (0.07)	0.26 (0.07)	0.31 (0.05)	0.24 (0.08)
GH-GNN	0.17 (0.03)	0.15 (0.02)	0.15 (0.02)	0.24 (0.14)	0.26 (0.06)	0.18 (0.06)
GH-GNN (pss)	**0.13 (0.02)**	**0.13 (0.02)**	**0.13 (0.03)**	**0.15 (0.05)**	**0.20 (0.07)**	**0.15 (0.05)**

	interpolation			extrapolation		
*R*^2^ ↑	*M*_n_	*M*_w_	*M*_n_/*M*_w_	*M*_n_	*M*_w_	*M*_n_/*M*_w_
random forest	0.72 (0.13)	0.79 (0.05)	0.69(0.17)	0.63 (0.23)	0.45 (0.35)	0.66 (0.15)
GH-GNN	0.90 (0.04)	0.91 (0.03)	0.92 (0.03)	0.66 (0.46)	0.64 (0.18)	0.86 (0.08)
GH-GNN (pss)	**0.94 (0.02)**	**0.94 (0.01)**	**0.94 (0.03)**	**0.90 (0.09)**	**0.81 (0.14)**	**0.89 (0.07)**

a The results are shown as the average performance across the 10 splits. The standard deviation is shown in between parentheses. The periodic unit polymer representation was used for all models. The arrow denotes what is “better” performance for each metric. The best average performance is shown in bold. The pre-trained and fine-tuned model is denoted by “pss”.

Remarkably, even though molar mass distribution information is absent for small-size systems, pretraining facilitates an enriched grasp of solute and solvent chemical structures and their corresponding activity coefficient values. This outcome underscores that transfer learning can be used to effectively leverage available information on small-size systems to improve the prediction of properties of polymer solutions.

### Discrete Interpolation

Table 2 includes the performance of the studied models in the task of interpolating within the polymer–solvent space defined by the data considered in this work. The random forest model achieves the poorest performance among the three models compared here. The modified GH-GNN model reduces the interpolation error consistently across all three data sets compared to that of the baseline. Furthermore, it reduces the prediction variability for doing interpolation as indicated by the standard deviation values in Table 2. This suggests that the modified GH-GNN model is able to achieve more consistent predictions despite the data on which the model was trained compared to the use of molecular fingerprints and relatively simple predictors. The performance and prediction consistency of the GH-GNN model for interpolation increase in practically all data sets when using transfer learning. We could not observe a significant advantage between the models using the number-average molar mass (*M*_n_), the weight-average molar mass (*M*_w_) or both. In all cases, the model achieves a similar performance for interpolation.

It is interesting to note that, during interpolation, the GH-GNN (pss) model achieves a similar MAE when predicting lnΩ_*ij*_^∞^ of polymer solutions as the one that the original GH-GNN model^20^ achieves when predicting small-size systems (i.e., around 0.13). This phenomenon may be attributed to the inherent limitations posed by the experimental uncertainty of logarithmic activity coefficients, which is estimated to lie within the range of 0.1–0.2.^43^ However, it is important to emphasize that this estimation of experimental uncertainty is rather generalized, and therefore, any conclusions drawn from this observation should be approached with caution.

### Solvent Extrapolation

Additionally, in Table 2, the models’ performance when extrapolating to previously unseen solvents is shown. An interesting observation emerges from comparing the, for instance, GH-GNN model’s average performance between interpolation and extrapolation scenarios. Notably, not only does accuracy diminish during extrapolation but also the prediction variability, indicated by the increase in standard deviation, becomes more pronounced. This phenomenon aligns with expectations, as extrapolating to new solvents within polymer–solvent mixtures presents a more challenging task compared to interpolating among known chemical species. This can be confirmed, by the fact that interpolation can even be approached (with remarkable accuracy) using matrix or tensor completion techniques that do not need explicit chemical structure insights.^43−45^

The same accuracy reduction from interpolation to extrapolation can be consistently observed in the case of the GH-GNN (pss) model. Furthermore, both modified GH-GNN models outperform the baseline model when extrapolating to other solvents, suggesting that the GNN-based models are more accurate not only when performing within the mixture space on which they were developed but also when extrapolating to unknown chemical species. Similar to the interpolation case, transfer learning consistently increases the accuracy and prediction reliability.

**Figure 6:**
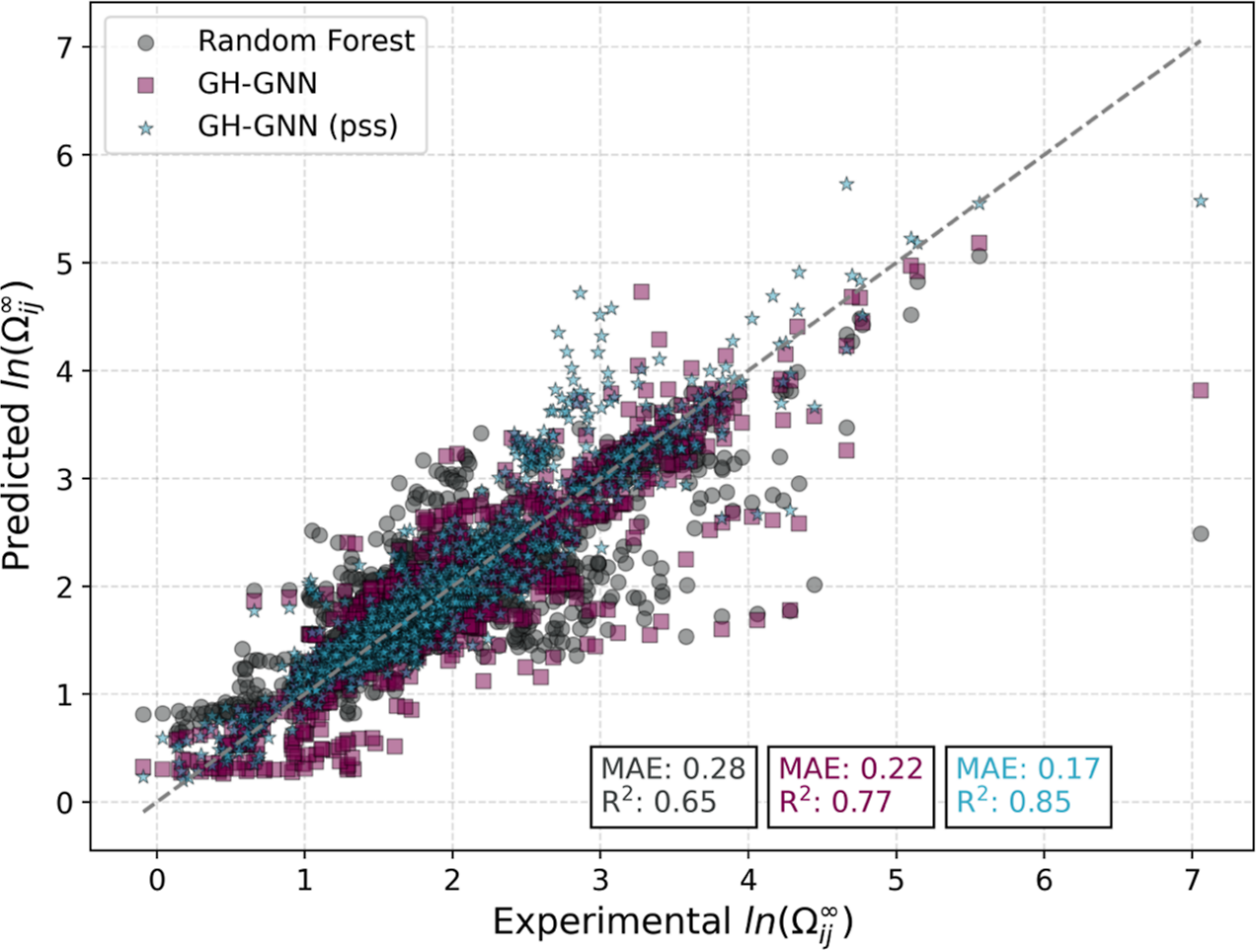
Parity performance achieved by the models in the extrapolation task on the *M*_w_ data set. The parity points are obtained as the average prediction across the 10 models generated with the 10 different splits (in an ensemble learning fashion). Only systems in the test set are included. The MAE and coefficient of determination (*R*^2^) are also given for the ensemble model. All models were trained using the periodic unit representation.

For illustration, the parity performance is shown in Figure 6 for the *M*_w_ data set. The parity points were obtained as the average prediction across the 10 models generated with the 10 different splits (in an ensemble learning fashion). Only systems in the test set are included. This means that if an observation has appeared, for instance, in four of the ten test sets, the final prediction is given as the average of the corresponding four models. All models were trained using the periodic unit representation. The first observation is that an ensemble model is able to improve the prediction accuracy of ln Ω_*ij*_^∞^ compared to a single model, as shown elsewhere^46,47^ for the prediction of ln γ_*ij*_^∞^. Specifically, the MAE of the GH-GNN model (pss) reduced from 0.20 to 0.17 for the ensemble prediction. Second, it is apparent that the spread of the parity points is significantly reduced in the case of the modified GH-GNN model compared to the random forest, and is even reduced further in the case of the GH-GNN (pss) model achieving an *R*^2^ of 0.85. This performance on solvent extrapolation is remarkable. However, as shown in our previous work,^20^ the estimation of the extrapolation performance should also consider an “extrapolation degree” metric (such as the Tanimoto indicator^20^) to account for the level of difficulty when extrapolating to a different solvent. It can be expected that as the extrapolation involves more distinct chemical species, the model performance will diminish.

### Phenomenological Models Comparison

The accuracy of the GH-GNN model was assessed by comparing it with the reported accuracy of the phenomenological models UNIFAC-ZM^16^ and Entropic-FV,^15^ as reported in the literature.^48^ The evaluation was based on the mean absolute percentage error (MAPE) of predicted Ω_*ij*_^∞^ values. For the GH-GNN predictions, we used the ensemble of pretrained and fine-tuned models for the extrapolation task and using the periodic unit representation. Only data points within the test set were included in the comparison. This implies that the GH-GNN model has not encountered the particular polymer–solvent combinations used here for a model comparison during its training. Furthermore, in the results presented for extrapolation, none of the solvents involved in the mixtures were part of any training combination. The *M*_n_ data set was given preference, if the test point was not part of the *M*_n_ data set, then the comparison was performed using the models trained on the *M*_w_ data set, and similarly for the *M*_n_/*M*_w_ data set in case the test point was neither part of the *M*_w_ data set. Table 3 shows the results of the comparison in the cases of GH-GNN interpolation and extrapolation.

**Table 3:** Comparison between the Pre-Trained and fine-tuned GH-GNN Model and the Phenomenological Models UNIFAC-ZM^16^ and Entropic-FV^15^ According to the MAPE of Ω_*ij*_^∞^^a^

systems	no. points	UNIFAC-ZM	Entropic-FV	GH-GNN (pss)
Interpolation				
athermal	51	12.0	10.3	**9.5**
polar	66	22.2	11.8	**8.7**
associated	21	27.9	33.8	**16.4**
Extrapolation				
athermal	53	11.1	9.3	**4.0**
polar	66	22.6	11.2	**6.4**
associated	21	27.9	33.8	**22.3**

a Best performance is shown in bold.

In the case of the phenomenological models, we utilized results obtained with temperature-dependent group contribution parameters, as these parameters were found to yield superior model performance.^48^ The comparison was conducted separately for athermal and polar systems, as well as systems involving hydrogen-bonding association, as outlined by Pappa et al.^48^ For the athermal case, the systems included polyethylene, polyethylene low-density, and polyisobutylene, along with linear, branched, and cyclic alkanes. In the case of polar systems, poly(ethyl methacrylate), poly(methyl acrylate), poly(methyl methacrylate), poly(*n*-butyl methacrylate), poly(vinyl acetate), polybutadiene, and polystyrene were examined, with ketones, esters, chlorinated hydrocarbons, benzene, and toluene as solvents. Regarding systems with associations, the set comprised poly(ethylene oxide), poly(methyl methacrylate), polybutadiene, and polystyrene, interacting with monohydroxy alcohols and acetic acid. Table 3 shows the performance comparison. A detailed description of the comparison for all systems studied here is provided in the Supporting Information.

Across all three system types, the GH-GNN (pss) model consistently achieved a lower MAPE in comparison to that of the phenomenological models. Nevertheless, it is important to note that the evaluation encompassed a limited set of data points, as outlined in Table 3. Consequently, any conclusions drawn from this comparison should be approached with caution, serving more as a stimulus for the potential directions of future research. It is also important to conduct this comparison with an awareness of the structurally distinct architectures of the different models. The UNIFAC-based models rely on pure-compound and binary interaction parameters of the groups within the mixture, whereas the GH-GNN model maintains a (significantly larger) fixed set of parameters regardless of the specific mixture being predicted. This structural difference prevents predictions for systems that cannot be fragmented into UNIFAC groups or where parameters are simply not available when using UNIFAC-based methods, while the GH-GNN model is structurally limited by the atomic and bond features involved during the model developing. A study of the applicability domain of the GH-GNN model can be performed similarly to the original publication^20^ using chemical classes or molecular similarity metrics. In line with this perspective, we hope to showcase the possibilities that machine learning, including GNN-based models, offers for advancing the field of polymer solution thermodynamic modeling.

## Conclusions

Machine learning models are significantly more prevalent in predicting properties of pure components compared with predicting properties of mixtures. Nonetheless, it is important to acknowledge the undeniable significance of mixtures in our everyday lives. Specifically, modeling the thermodynamic behavior of polymer–solvent mixtures is of high relevance when designing and optimizing polymer industrial processes. In this work, we have proposed a simple modification of the GH-GNN model^20^ for predicting activity coefficients at infinite dilution of polymer solutions Ω_*ij*_^∞^. The performance of this modified GH-GNN model was studied using a data set of experimentally determined Ω_*ij*_^∞^ values. The performance of different polymer representations (including monomer(s), repeating unit, periodic unit, and oligomer) was evaluated in terms of prediction accuracy. The finding suggests that in low-data regimes, such in the case of polymer–solvent mixtures, the exact polymer representation is less influential than in regimes of large data availability. However, the intrinsic drawbacks of some representations are undeniable and should be considered despite the amount of data at hand. The curated data set used here augmented with the corresponding SMILES of all solvents and polymers (with all considered representations) is provided as a benchmark to incentive research in this direction.

Moreover, transfer learning was used to leverage the more abundant activity coefficient data available for small-size systems compared to the data analogous in polymer solutions. As a result, not only a significant increase in the model’s accuracy was observed but also a reduction in the predictions variability. This suggests that transfer learning is also useful for increasing the consistency, and hence reliability, of the predictions. The proposed modified GH-GNN model achieved remarkable performance in both interpolation and extrapolation tasks, opening the realm of using recent advances in machine learning for polymer mixture property prediction. Finally, a comparison between the modified GH-GNN model and the UNIFAC-ZM^16^ and Entropic-FV^15^ models was provided showing promising potential in the case of the proposed GNN-based model.

The inclusion of copolymers as part of the training and evaluation of the models is left as a future research direction. Furthermore, the generalization of GH-GNN to multicomponent systems can be directly implemented in the current framework considering that a single molecular-level GNN is able to process all molecular graphs, and a single mixture-GNN is able to process the mixture graph despite the number of nodes and edges. Nonetheless, the best approach for incorporating mixture composition remains an unresolved question. Moreover, studying the performance of COSMO-based models in comparison with machine learning-based models in the context of polymer solutions remains an open question. Capturing more complex polymer distributions that cannot be completely characterized by a single average molar mass metric is still a challenge. This is specially important in the case of biobased polymers like lignin involving much more complex molar mass distributions.^49^ However, the future role of this type of biopolymeric materials is envisioned to become increasingly important in the context of biorefineries.

## Supporting Information Available

Pretraining loss plots. Description of the errors corrected from the original DECHEMA collection. Percentage of distinct polymers and solvents in each data set. Temperature and molecular mass descriptors (i.e., *M*_n_ and *M*_w_) distributions. Performance of polymer representations across all data sets, models and tasks. Detailed description of the systems considered in the phenomenological vs GH-GNN comparison (PDF)

## Notes

Published as part of *The Journal of Physical Chemistry A*virtual special issue “Machine Learning in Physical Chemistry Volume 2”.
