# Supplementary material for: Gibbs–Helmholtz Graph Neural Network for the Prediction of Activity Coefficients of Polymer Solutions at Infinite Dilution
Source: J Phys Chem A. 2023 Nov 9;127(46):9863–73. doi: 10.1021/acs.jpca.3c05892 (PMC10683018; doi:10.1021/acs.jpca.3c05892)
Supplement: Supplementary file 1 — jp3c05892_si_001.pdf [file jp3c05892_si_001.pdf]

Supporting Information for

A Gibbs-Helmholtz Graph Neural Network for the Prediction of Activity Coefficients of Polymer Solutions at Infinite Dilution

Edgar Ivan Sanchez Medina[1], Sreekanth Kunchapu[1] and Kai Sundmacher[1,2,*]
[1] Chair for Process Systems Engineering, Otto-von-Guericke University,
Universitätsplatz 2, Magdeburg, 39106, Germany
[2] Process Systems Engineering, Max Planck Institute for Dynamics of Complex Technical Systems,
Sandtorstraße 1, Magdeburg, 39106, Germany
[*]Corresponding author. E-mail: sundmacher@mpi-magdeburg.mpg.de

October 2023

S1 Pre-training loss function evolution

Figures S1 and S2 show the evolution of the train and validation loss during the pre-training of the GH-GNN model on small-size systems for the case of extra features MN and MN and MW, respectively.

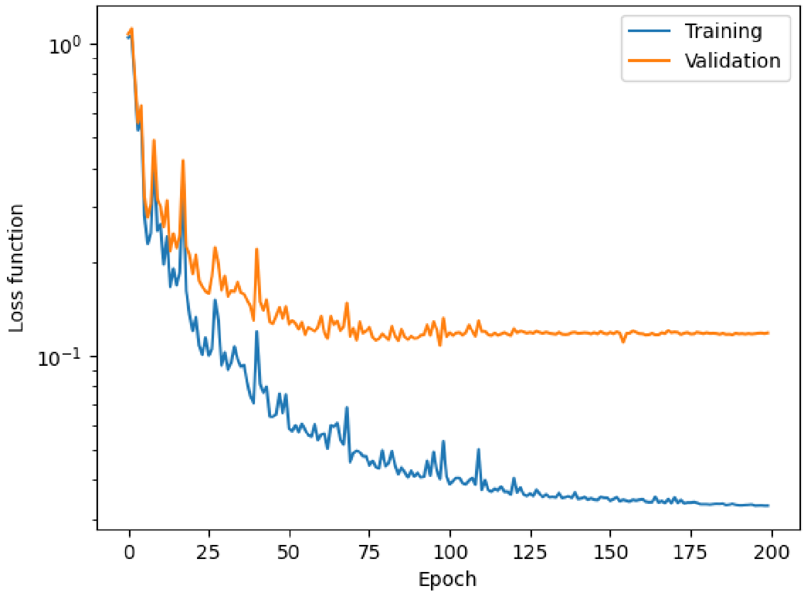

Figure S1: Train and validation convergence during the pre-training of the GH-GNN model on small-size systems using MN as extra global-level feature.

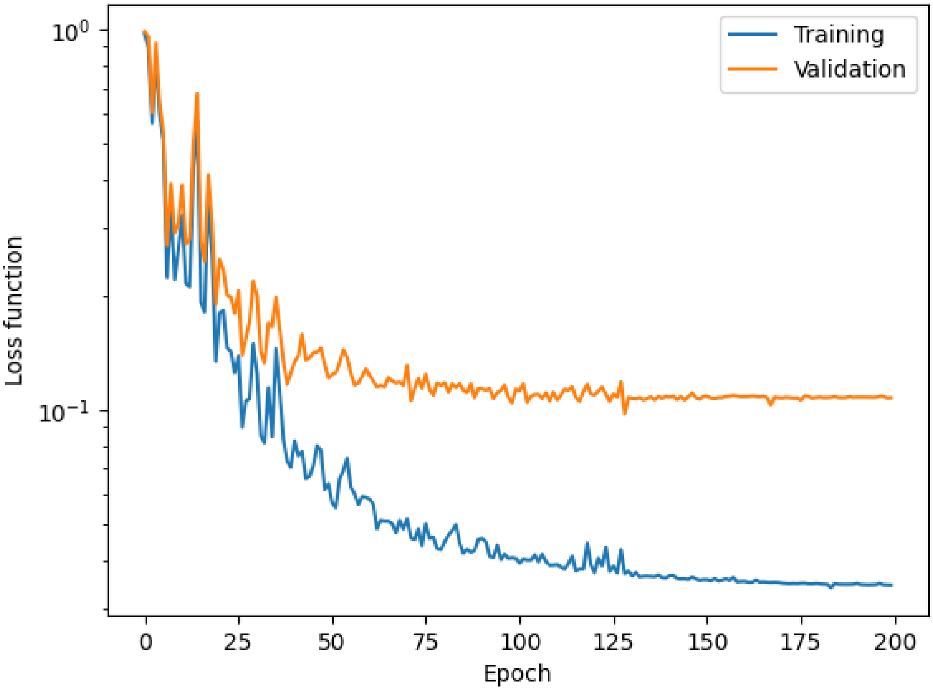

Figure S2: Train and validation convergence during the pre-training of the GH-GNN model on small-size systems using MN and MW as extra global-level features.

S2 Corrections to the original DECHEMA collection of polymer solution data

The following errors were detected on the original data collection of activity coefficients at infinite dilution for polymer solutions contained in volume XIV of the DECHEMA Chemistry Data Series [1]. These errors have been corrected in our curated data set.

- The chemical formula of glycerol triacetate is shown as $C_9H_{15}O_6$. However, the correct formula should be $C_9H_{14}O_6$
- The name of the polymer referred to as " poly(ϵ-valerolactone)" should be "poly(δ-valerolactone) according to the original paper from where the data was taken from [3]. In Table 1 of this paper the above mentioned typo occurred. Therefore, it is likely that the error propagated from there to the DECHEMA collection.
- The polymer "polyehtylene low-density" has been now annotated as a branched polymer using note 86.
- The polymer "polystyrene, antishock" is shown in the original DECHEMA data collection as a homopolymer. However, this is a copolymer also known as "high impact polystyrene (HIPS)".
- The original data collection show the polymers "polyoxyethylene, α,ω-dihydroxy" and "poly(ethylene oxide)" as two distinct polymers. However, while one specifies the start and end groups, it is very likely that both refer to the same structure.

S3 Composition of polymers and solvents in each data set

Figures in this section show the percentage composition of polymers and solvents in each data set.

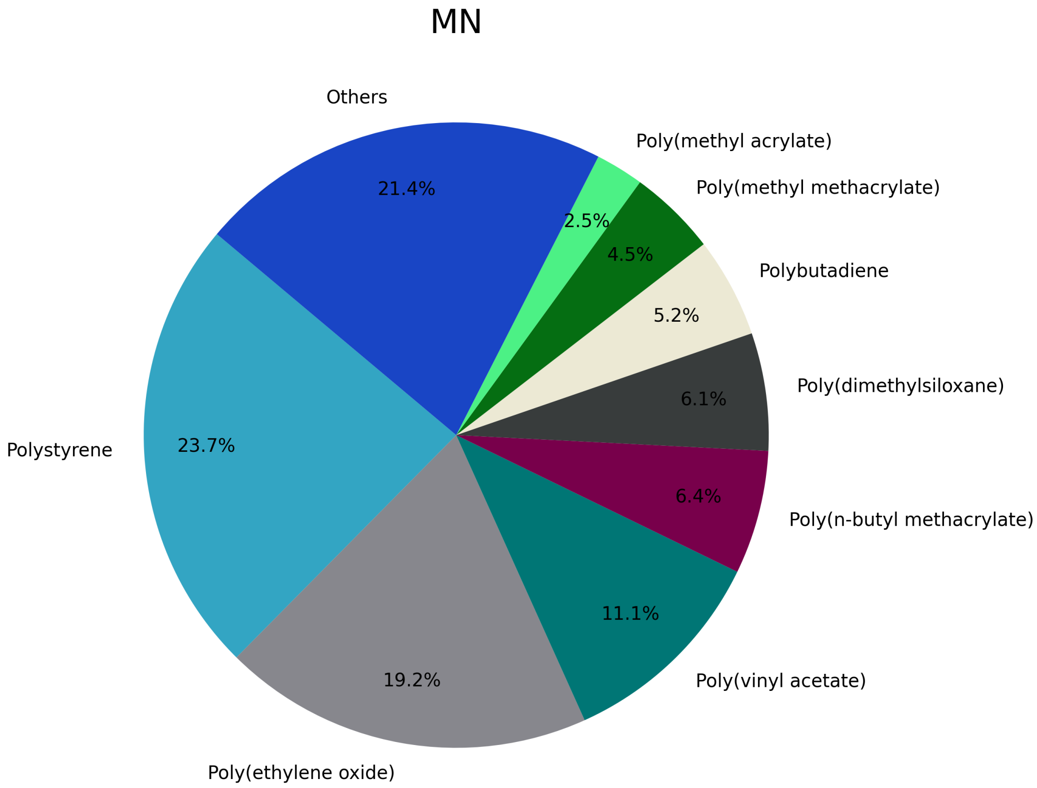

Figure S3: Percentage composition of polymers in the MN data set.

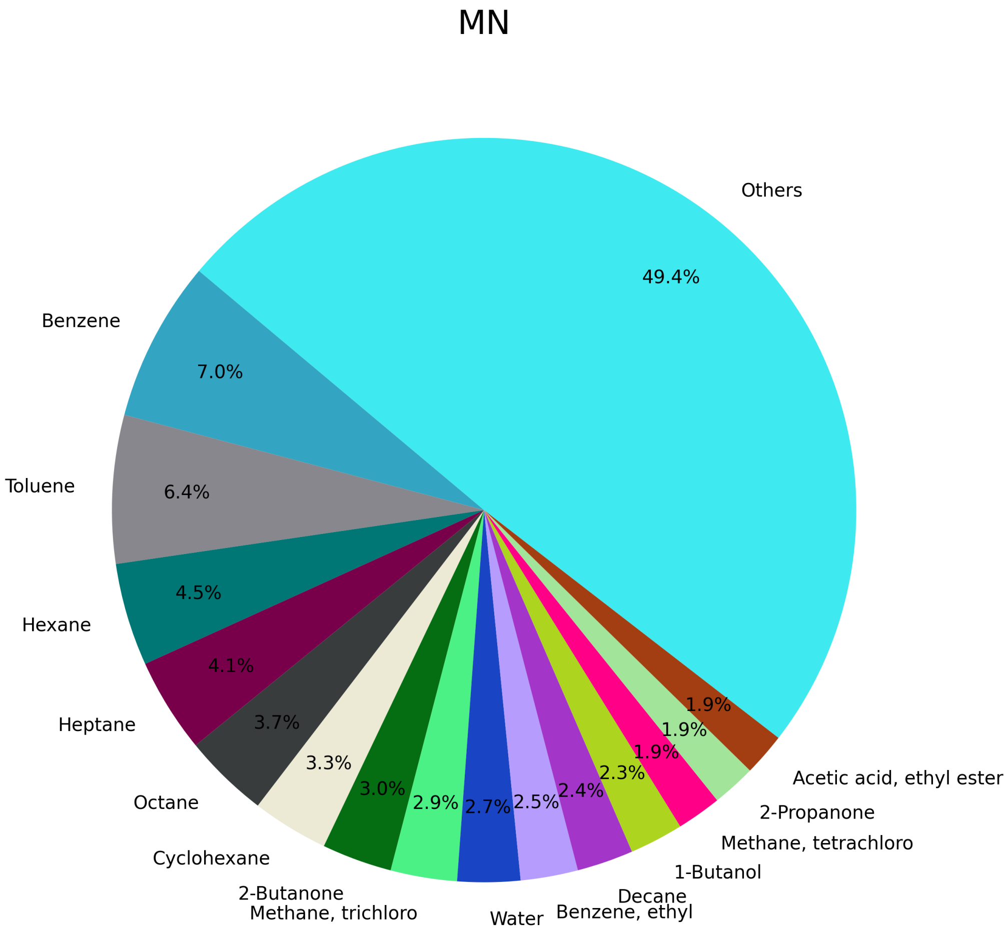

Figure S4: Percentage composition of solvents in the MN data set.

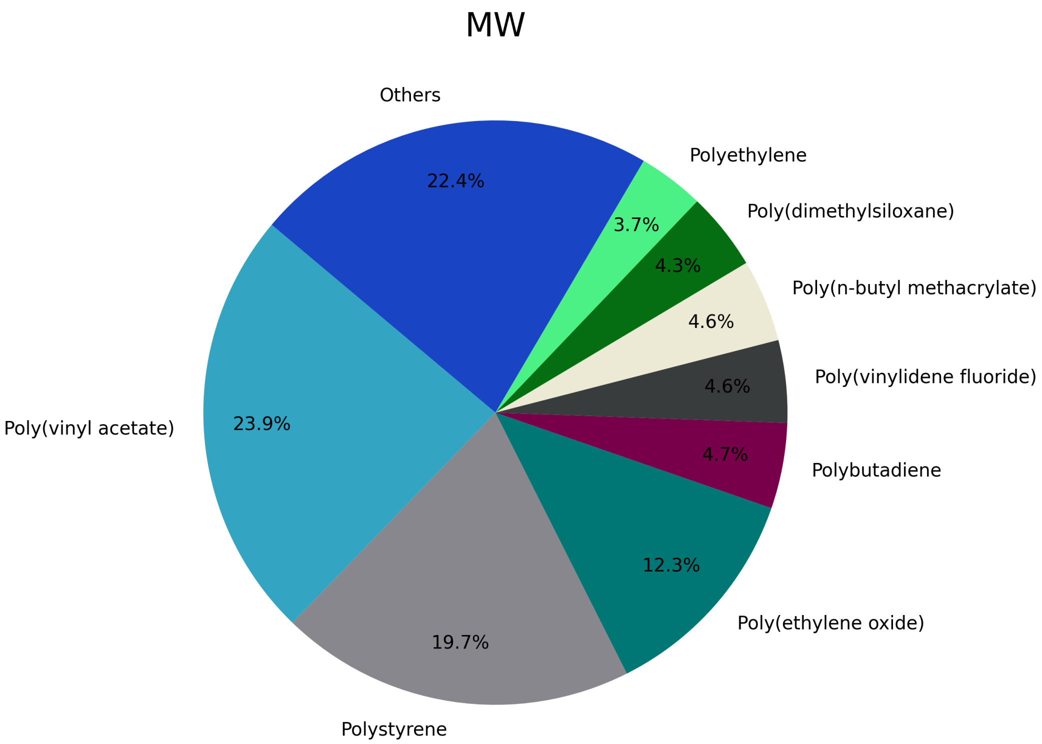

Figure S5: Percentage composition of polymers in the MW data set.

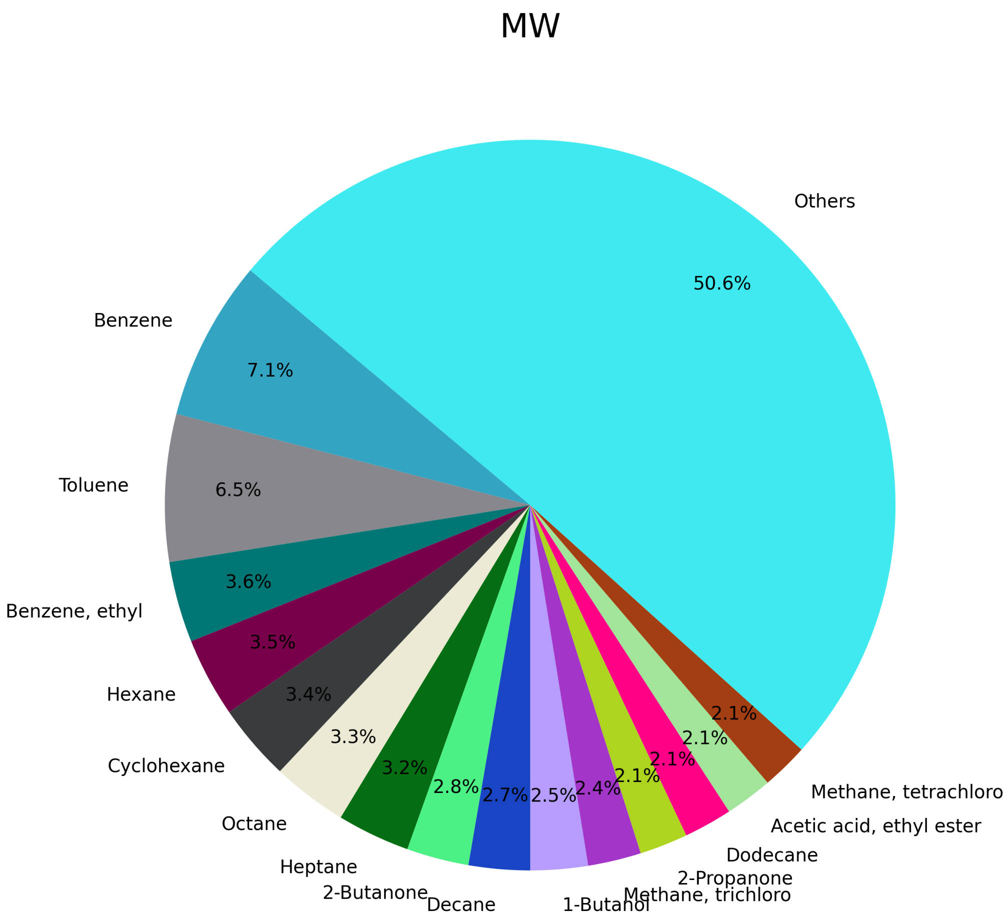

Figure S6: Percentage composition of solvents in the *MW* data set.

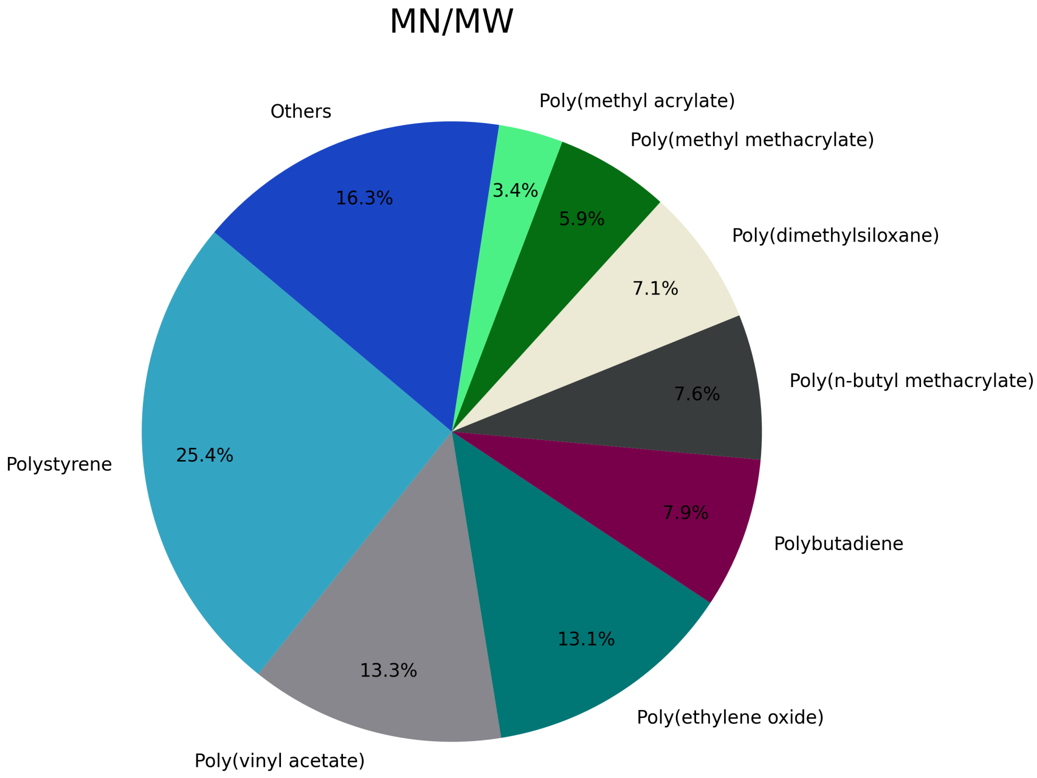

Figure S7: Percentage composition of polymers in the MN/MW data set.

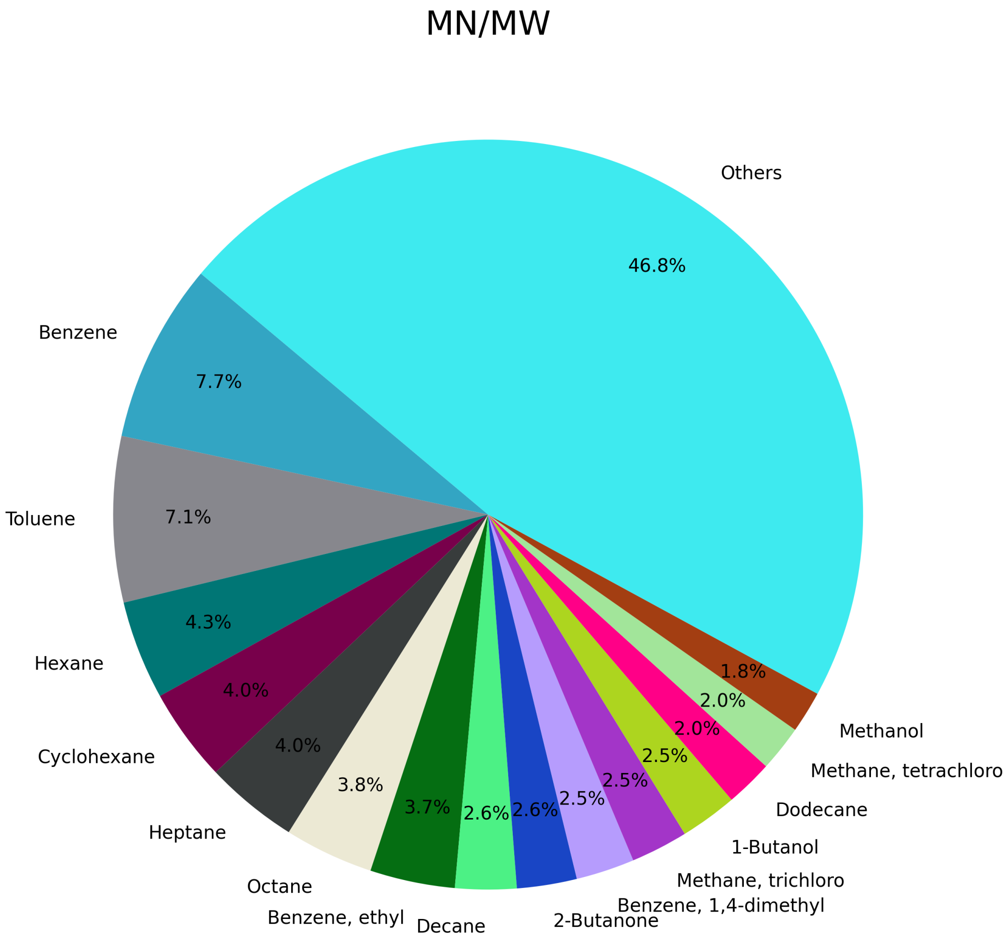

Figure S8: Percentage composition of solvents in the MN/MW data set.

S4 Temperature, activity coefficient and polymer molecular mass descriptors distributions

The figures in this section show the distribution of the temperature, $\ln \Omega_{ij}^{\infty}$ and molar mass distribution descriptors of each data set.

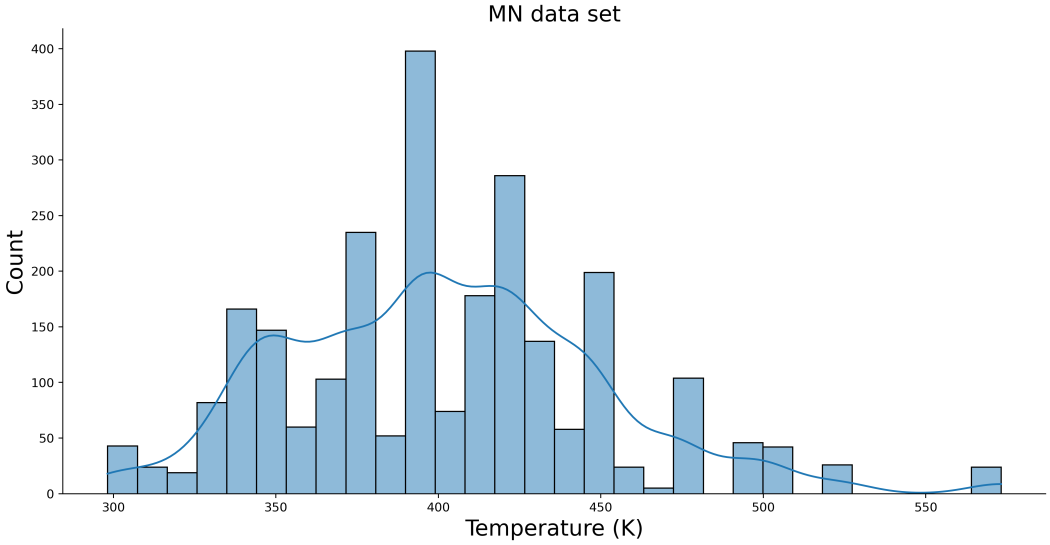

Figure S9: Distribution of temperature in the MN data set.

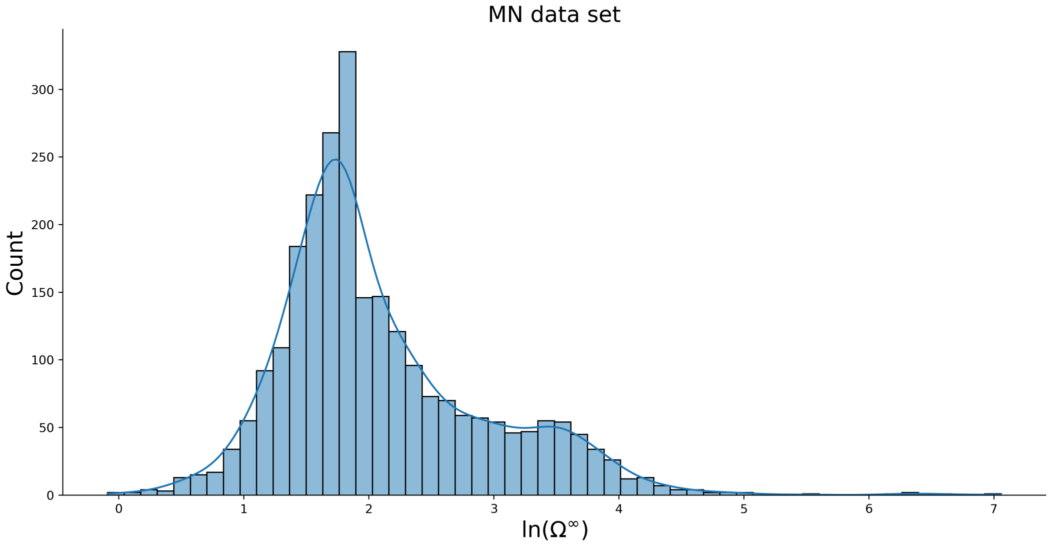

Figure S10: Distribution of $\ln \Omega_{ij}^{\infty}$ temperature in the MN data set.

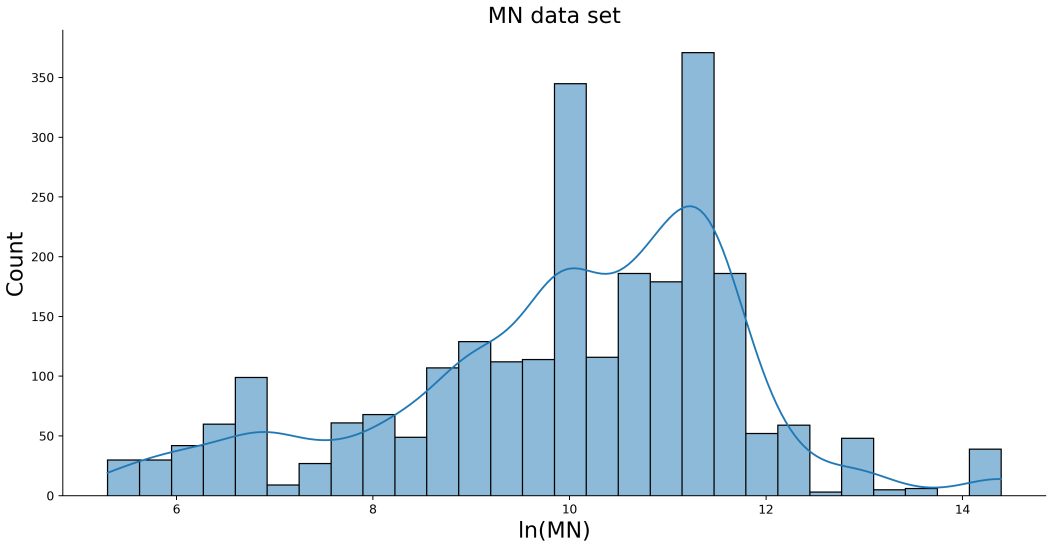

Figure S11: Distribution of $\ln MN$ temperature in the MN data set.

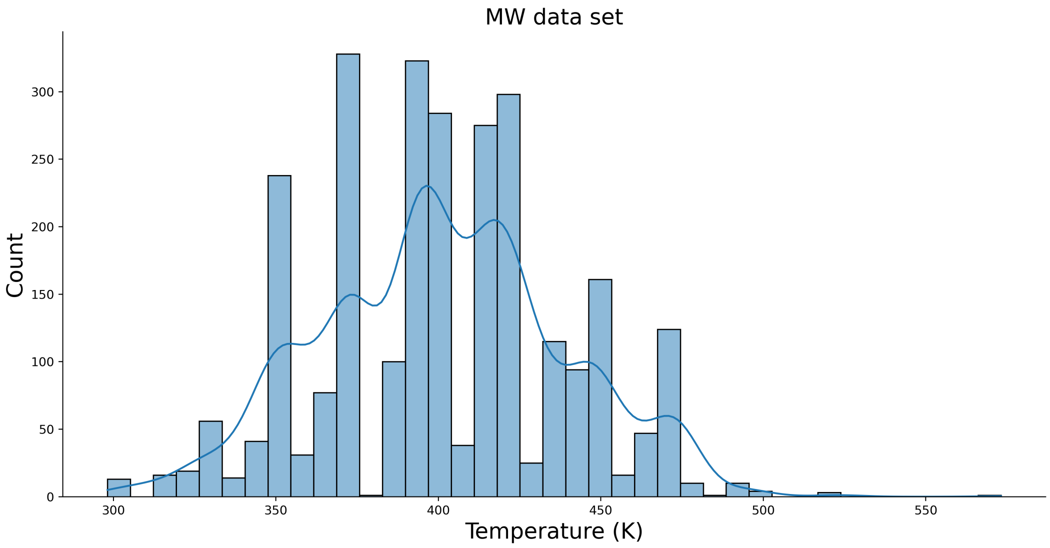

Figure S12: Distribution of temperature in the MW data set.

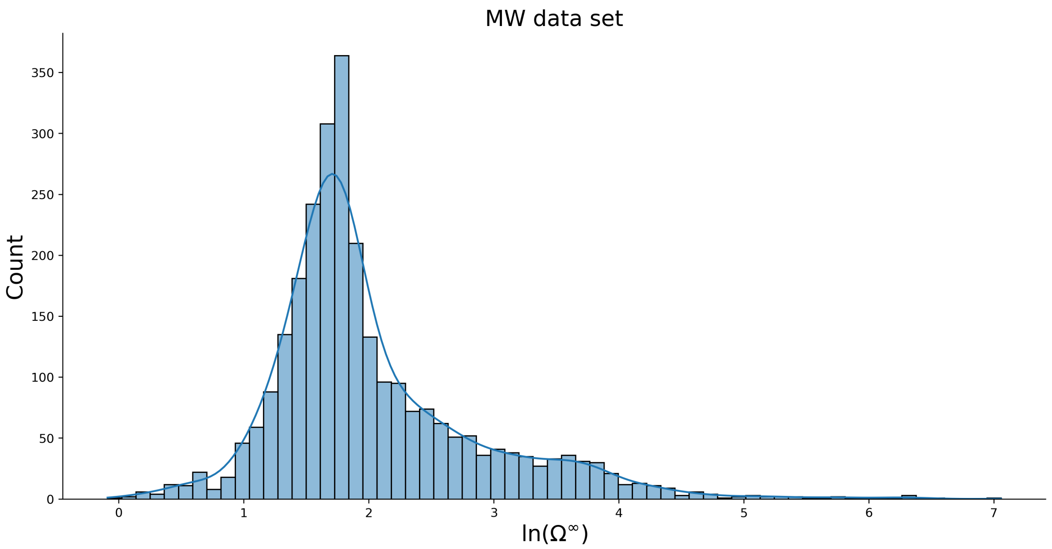

Figure S13: Distribution of $\ln \Omega_{ij}^{\infty}$ temperature in the MW data set.

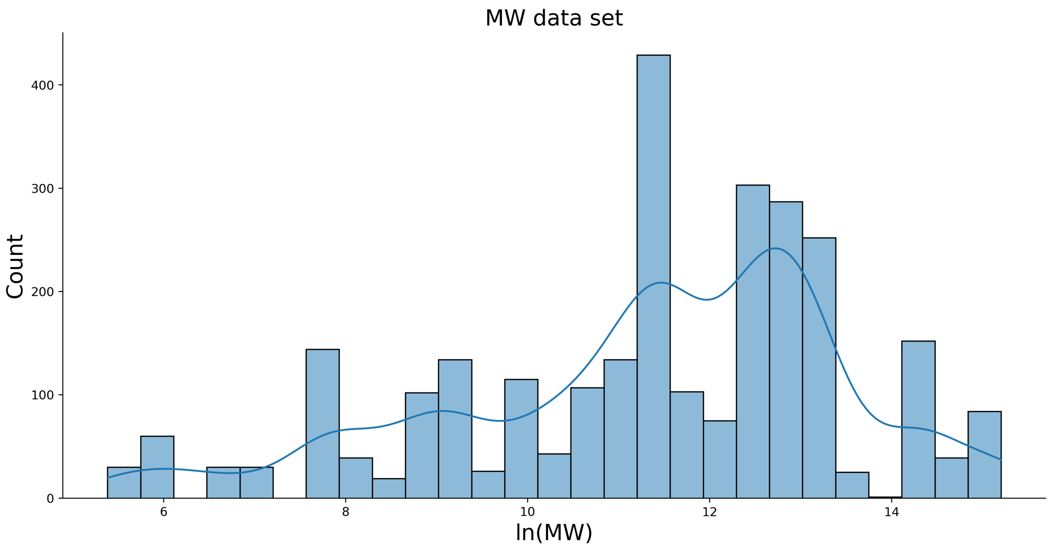

Figure S14: Distribution of $\ln MW$ temperature in the MW data set.

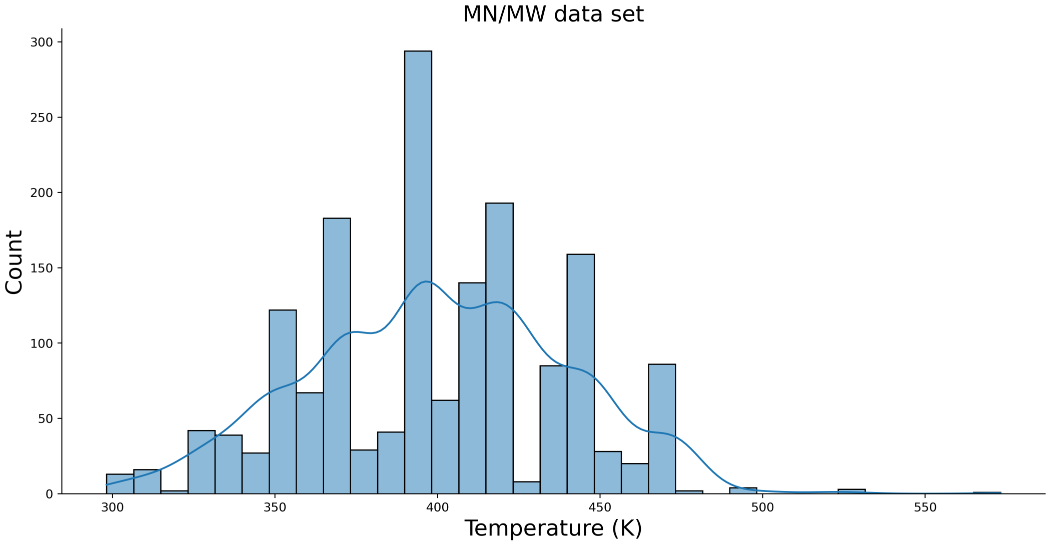

Figure S15: Distribution of temperature in the MN/MW data set.

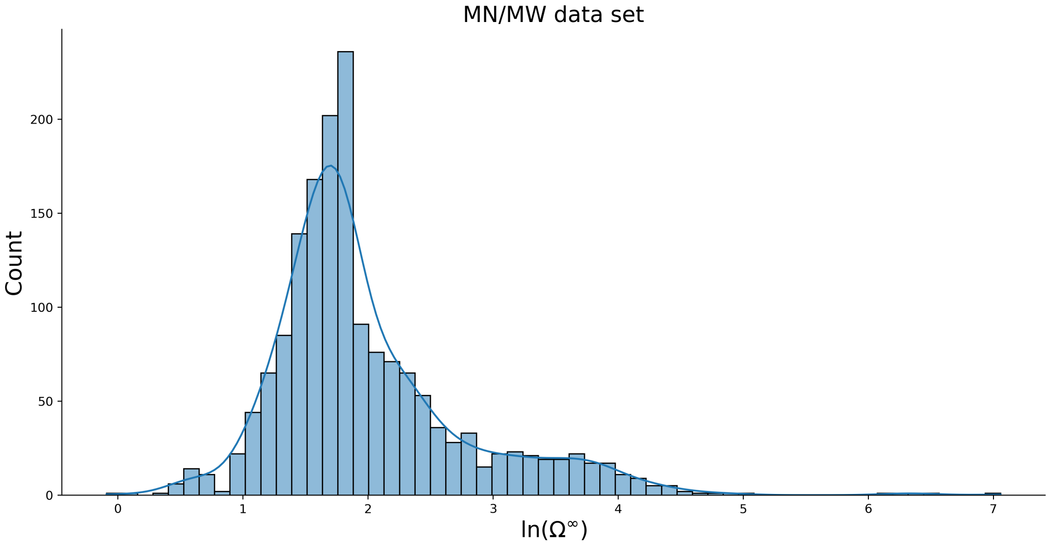

Figure S16: Distribution of $\ln \Omega_{ij}^{\infty}$ temperature in the MN/MW data set.

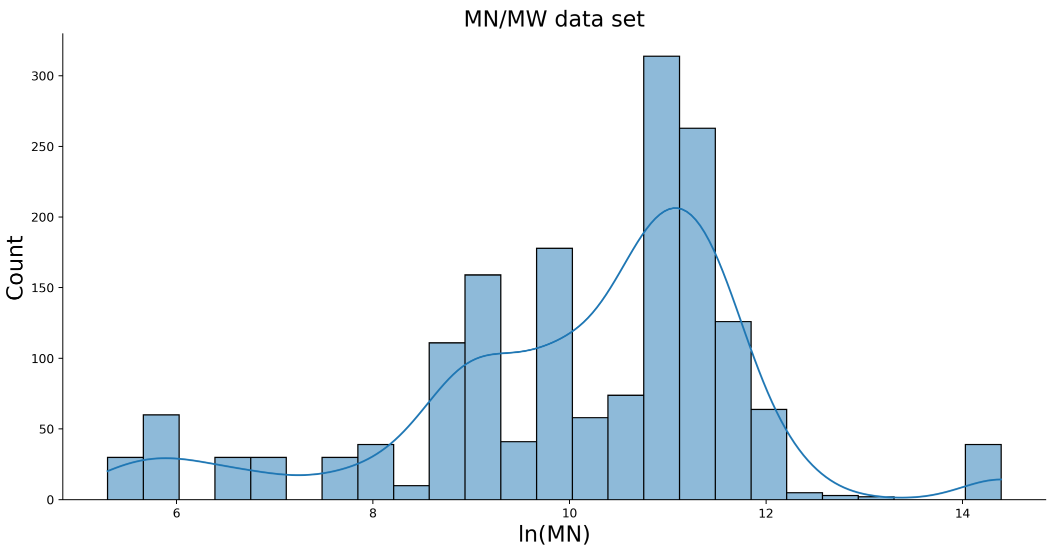

Figure S17: Distribution of $\ln MN$ temperature in the MN/MW data set.

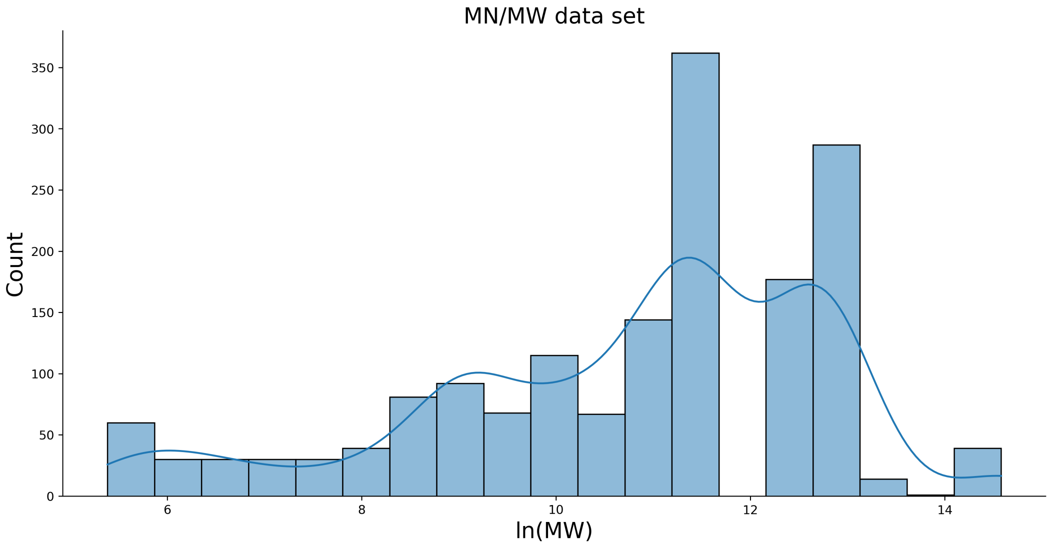

Figure S18: Distribution of $\ln MW$ temperature in the MN/MW data set.

S5 Performance of polymer representations across all data sets, models for interpolation

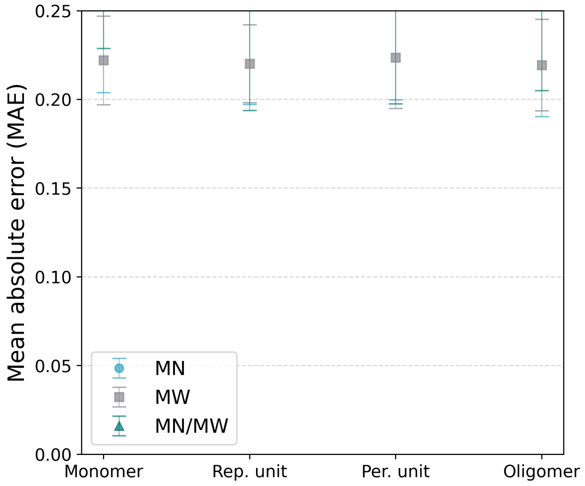

Figure S19: Performance of the random forest baseline according to the mean absolute error on the test set for the interpolation task.

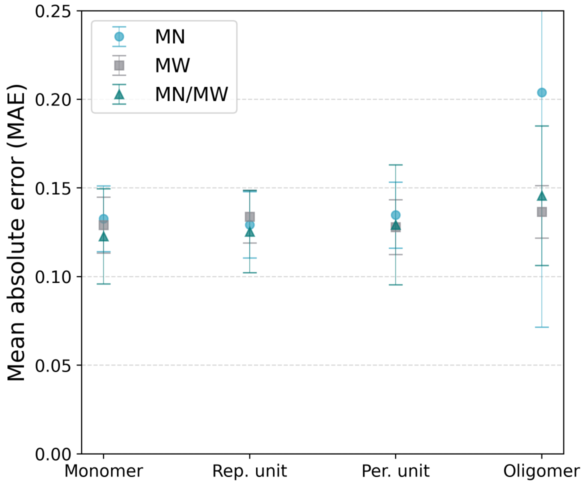

Figure S20: Performance of the pre-trained and fine-tuned GH-GNN model according to the mean absolute error on the test set for the interpolation task.

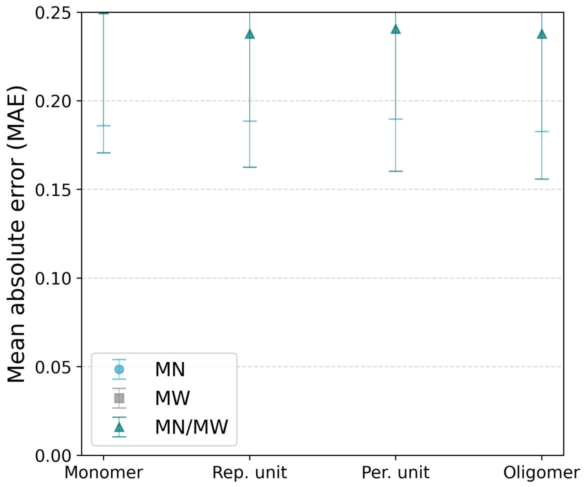

Figure S21: Performance of the random forest baseline according to the mean absolute error on the test set for the extrapolation task.

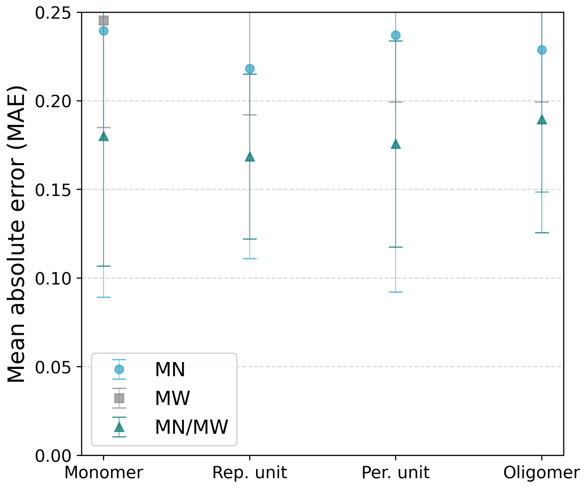

Figure S22: Performance of the modified GH-GNN model (without pre-training) according to the mean absolute error on the test set for the extrapolation task.

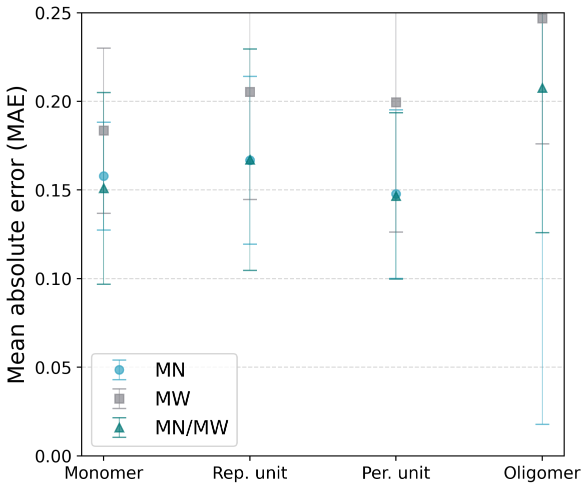

Figure S23: Performance of the pre-trained and fine-tuned GH-GNN model according to the mean absolute error on the test set for the extrapolation task.

S6 Systems considered in the phenomenological vs. GH-GNN comparison

Table S1: Comparison between the pre-trained GH-GNN model and the phenomenological models UNIFAC-ZM and Entropic-FV according to the mean absolute percentage error of Ω_{ij}^{∞} on the athermal systems. All GH-GNN models here correspond to the ones tested for interpolation. "G-" denotes the GH-GNN model trained on the corresponding data set. The last column corresponds to the reported performance as in the manuscript. The predictions from the phenomenological models were taken from the literature [2]. "#" denotes the number of points.

Solute	Solvent	MN	MW	T_1	T_2	#	UNIFAC-ZM	Entropic-FV	G-MN	G-MW	G-MN/MW	GH-GNN
Polyethylene, low-density	Cyclohexane	35000	235000	383.2	473.2	5	3.9	4.9	11.0	18.6	16.9	11.0
Polyethylene, low-density	Hexane	35000	235000	383.2	473.2	5	20.6	7.9	2.6	4.6	2.5	2.6
Polyethylene, low-density	Heptane	35000	235000	383.2	473.2	5	12.6	6.1	2.1		0.7	2.1
Polyethylene, low-density	Octane	35000	235000	383.2	473.2	5	6.6	5.6	7.4	0.7	1.1	7.4
Polyethylene	Butane	16600	82300	397.2	423.2	2	33.9	35.9	8.1			8.1
Polyethylene	Hexane	15000		413	443	2	23.3	7	10.7			10.7
Polyethylene	Hexane	16600	82300	397.2	473.2	3	14.5	10.4	1.1	5.8	5.6	1.1
Polyethylene	Octane	15000		413	443	2	22	24	25.7			25.7
Polyethylene	Octane	16600	82300	397.2	523.2	4	2	10.7	2.8	9.2	9.6	2.8
Polyethylene	Octane		82000	393.2	418.3	2	4.1	5.8		1.7		1.7
Polyethylene	Octane		105000	418.6	425.8	2	7.3	8.5		4.3		4.3
Polyethylene	Nonane		82000	393.2	418.3	2	0.8	6.5		7.0		7.0
Polyethylene	Nonane		105000	418.6	425.8	2	6.1	10.1		3.2		3.2
Polyethylene	Decane		82000	393.2	418.3	2	9.3	1		4.1		4.1
Polyethylene	Decane		105000	418.6	426.5	2	2.6	9.9		1.3		1.3
Polyethylene	Dodecane		82000	393.2	418.3	2	7.9	7.4		36.1		36.1
Polyethylene	Dodecane		105000	418.6	426.5	2	2.6	10.6		32.2		32.2
Polyisobutylene	Pentane	860000		313.1	323.1	2	36.4	13.6	9.3			9.3

Table S2: Comparison between the pre-trained GH-GNN model and the phenomenological models UNIFAC-ZM and Entropic-FV according to the mean absolute percentage error of Ω_{ij}^{∞} on the athermal systems. All GH-GNN models here correspond to the ones tested for extrapolation. "G-" denotes the GH-GNN model trained on the corresponding data set. The last column corresponds to the reported performance as in the manuscript. The predictions from the phenomenological models were taken from the literature [2]. "#" denotes the number of points.

Solute	Solvent	MN	MW	T_1	T_2	#	UNIFAC-ZM	Entropic-FV	G-MN	G-MW	G-MN/MW	GH-GNN
Polyethylene, low-density	Cyclohexane	35000	235000	383.2	473.2	5	3.9	4.9		20.0	14.2	14.2
Polyethylene, low-density	Hexane	35000	235000	383.2	473.2	5	20.6	7.9			4.2	4.2
Polyethylene, low-density	Heptane	35000	235000	383.2	473.2	5	12.6	6.1	0.9			0.9
Polyethylene, low-density	Octane	35000	235000	383.2	473.2	5	6.6	5.6		3.5		3.5
Polyethylene	Butane	16600	82300	397.2	423.2	2	33.9	35.9	7.4	3.3		7.4
Polyethylene	Hexane	16600	82300	397.2	473.2	3	14.5	10.4			2.6	2.6
Polyethylene	Hexane, 3-methyl		82000	393.2	418.3	2	7.9	6.4		7.4		7.4
Polyethylene	Hexane, 3-methyl		105000	418.6	425.8	2	18	14.1		1.5		1.5
Polyethylene	Heptane, 3-methyl		82000	393.2	418.3	2	10.1	4.2		1.0		1.0
Polyethylene	Heptane, 3-methyl		105000	418.6	425.8	2	16.2	8.6		2.3		2.3
Polyethylene	Hexane, 2,4-dimethyl		82000	393.2	418.3	2	7.4	5.4		1.3		1.3
Polyethylene	Hexane, 2,4-dimethyl		105000	418.6	425.8	2	12.2	7.6		1.8		1.8
Polyethylene	Hexane, 2,5-dimethyl		82000	393.2	418.3	2	10.5	8		0.2		0.2
Polyethylene	Hexane, 2,5-dimethyl		105000	418.6	425.8	2	15.4	10.4		3.9		3.9
Polyethylene	Octane	16600	82300	397.2	523.2	4	2	10.7		11.2		11.2
Polyethylene	Octane		82000	393.2	418.3	2	4.1	5.8		1.6		1.6
Polyethylene	Octane		105000	418.6	425.8	2	7.3	8.5		2.2		2.2
Polyethylene	Nonane		82000	393.2	418.3	2	0.8	6.5		6.5		6.5
Polyethylene	Nonane		105000	418.6	425.8	2	6.1	10.1		2.4		2.4

Table S3: Comparison between the pre-trained GH-GNN model and the phenomenological models UNIFAC-ZM and Entropic-FV according to the mean absolute percentage error of Ω_{ij}^{∞} on the polar systems. All GH-GNN models here correspond to the ones tested for interpolation. "G-" denotes the GH-GNN model trained on the corresponding data set. The last column corresponds to the reported performance as in the manuscript. The predictions from the phenomenological models were taken from the literature [2]. "#" denotes the number of points.

Solute	Solvent	MN	MW	T_1	T_2	#	UNIFAC-ZM	Entropic-FV	G-MN	G-MW	G-MN/MW	GH-GNN
Poly(vinyl acetate)	Acetic acid, ethyl ester	83350	331400	398.2	473.2	4	18	5	3.5		3.3	3.5
Polybutadiene	Acetic acid, ethyl ester	22600	23956	353	373	3	7	2.8			4.5	4.5
Poly(methyl methacrylate)	Acetic acid, methyl ester	85100	200000	398.5	434.2	4	36.5	6.8	11.0	6.8		11.0
Poly(ethyl methacrylate)	Acetic acid, ethyl ester	40000		393.2		1	37.9	18.8	3.2			3.2
Poly(n-butyl methacrylate)	Acetic acid, ethyl ester	8716	9239	373	413	3	25.9	18.6	9.2	4.0	9.1	9.2
Poly(vinyl acetate)	2-Butanone	83350	331400	398.2	473.2	4	3.3	17.7	5.3			5.3
Polystyrene	2-Butanone	96200	97600	396.5	447.5	3	14.3	9.7			16.2	16.2
Polybutadiene	2-Butanone	93000	99000	339.2	369.2	4	39.1	22.9	2.3	4.5		2.3
Polybutadiene	2-Pentanone, 4-methyl	22600	23956	353	373	3	18.6	2.5	15.9	2.7	17.3	15.9
Polybutadiene	2-Propanone	22600	23956	353	373	3	14.2	10.1	16.7	11.4	5.4	16.7
Poly(n-butyl methacrylate)	2-Propanone	8716	9239	373	413	3	19.6	14.4	4.4	12.8		4.4
Poly(ethyl methacrylate)	2-Propanone	144000		417.7		1	22.9	31	1.8			1.8
Poly(n-butyl methacrylate)	Butane, 1-chloro	73500	320000	393.2	413.2	3	15.1	17.4			3.0	3.0
Poly(methyl methacrylate)	Methane, dichloro	6107	6412	423	473	3	47.5	19	13.0		2.6	13.0
Poly(n-butyl methacrylate)	Methane, dichloro	73500	320000	393.2	413.2	3	21.4	9.3	7.9	1.2		7.9
Polystyrene	Benzene, chloro	96200	97600	396.5	447.5	3	32.5	9.9		22.5		22.5
Polybutadiene	Methane, tetrachloro	22600	23956	353	373	3	8.9	7.1	9.5	4.5	4.0	9.5
Poly(methyl acrylate)	Benzene	63200	200000	363.2	383.2	3	28.2	5.6	12.2			12.2
Polybutadiene	Benzene	22600	23956	353	373	3	10	5.7	14.9	7.8		14.9
Poly(methyl acrylate)	Toluene	63200	200000	363.2	383.2	3	21	6.8	2.6			2.6
Polystyrene	Toluene	76000	82000	413.2	473.2	5	13.8	5.9	7.5	4.4		7.5
Poly(ethyl methacrylate)	Toluene	144000		417.7		1	31.9	13	3.2			3.2

Table S4: Comparison between the pre-trained GH-GNN model and the phenomenological models UNIFAC-ZM and Entropic-FV according to the mean absolute percentage error of Ω_{ij}^{∞} on the polar systems. All GH-GNN models here correspond to the ones tested for extrapolation. "G-" denotes the GH-GNN model trained on the corresponding data set. The last column corresponds to the reported performance as in the manuscript. The predictions from the phenomenological models were taken from the literature [2]. "#" denotes the number of points.

Solute	Solvent	MN	MW	T_1	T_2	#	UNIFAC-ZM	Entropic-FV	G-MN	G-MW	G-MN/MW	GH-GNN
Poly(vinyl acetate)	Acetic acid, ethyl ester	83350	331400	398.2	473.2	4	18	5	3.1	9.5	8.5	3.1
Polybutadiene	Acetic acid, ethyl ester	22600	23956	353	373	3	7	2.8	10.6	10.0	2.4	10.6
Poly(methyl methacrylate)	Acetic acid, methyl ester	85100	200000	398.5	434.2	4	36.5	6.8	11.2	12.1	7.2	11.2
Poly(ethyl methacrylate)	Acetic acid, butyl ester	144000		417.7		1	33	17	6.9			6.9
Poly(ethyl methacrylate)	Acetic acid, ethyl ester	40000		393.2		1	37.9	18.8	5.2			5.2
Poly(n-butyl methacrylate)	Acetic acid, ethyl ester	8716	9239	373	413	3	25.9	18.6	7.5	3.8	15.5	7.5
Poly(vinyl acetate)	2-Butanone	83350	331400	398.2	473.2	4	3.3	17.7	1.8	13.6	9.3	1.8
Polystyrene	2-Butanone	96200	97600	396.5	447.5	3	14.3	9.7	5.2	14.2	8.6	5.2
Polybutadiene	2-Butanone	93000	99000	339.2	369.2	4	39.1	22.9	11.9	15.5	22.8	11.9
Polybutadiene	2-Pentanone, 4-methyl	22600	23956	353	373	3	18.6	2.5	6.3		13.4	6.3
Polybutadiene	2-Propanone	22600	23956	353	373	3	14.2	10.1		8.1	6.6	6.6
Poly(n-butyl methacrylate)	2-Propanone	8716	9239	373	413	3	19.6	14.4		2.6	16.5	16.5
Poly(n-butyl methacrylate)	Butane, 1-chloro	73500	320000	393.2	413.2	3	15.1	17.4	2.8	1.9	1.4	2.8
Poly(methyl methacrylate)	Methane, dichloro	6107	6412	423	473	3	47.5	19	2.6		8.1	2.6
Poly(n-butyl methacrylate)	Methane, dichloro	73500	320000	393.2	413.2	3	21.4	9.3	2.8		2.8	2.8
Polystyrene	Benzene, chloro	96200	97600	396.5	447.5	3	32.5	9.9	12.9	24.3		12.9
Polybutadiene	Methane, tetrachloro	22600	23956	353	373	3	8.9	7.1	3.6	21.8	31.5	3.6
Poly(methyl acrylate)	Benzene	63200	200000	363.2	383.2	3	28.2	5.6	9.1	1.4		9.1
Polybutadiene	Benzene	22600	23956	353	373	3	10	5.7	4.8	12.7		4.8
Poly(methyl acrylate)	Toluene	63200	200000	363.2	383.2	3	21	6.8	7.7	7.1	4.5	7.7
Polystyrene	Toluene	76000	82000	413.2	473.2	5	13.8	5.9	0.9	2.1	7.1	0.9
Poly(ethyl methacrylate)	Toluene	144000		417.7		1	31.9	13	2.0			2.0

Table S5: Comparison between the pre-trained GH-GNN model and the phenomenological models UNIFAC-ZM and Entropic-FV according to the mean absolute percentage error of Ω_{ij}^{∞} on the systems with association. All GH-GNN models here correspond to the ones tested for interpolation. "G-" denotes the GH-GNN model trained on the corresponding data set. The last column corresponds to the reported performance as in the manuscript. The predictions from the phenomenological models were taken from the literature [2]. "#" denotes the number of points.

Solute	Solvent	MN	MW	T_1	T_2	#	UNIFAC-ZM	Entropic-FV	G-MN	G-MW	G-MN/MW	GH-GNN
Poly(ethylene oxide)	1-Butanol	2000	2180	348.2	398.2	3	39.1	15.1	12.0		29.3	12.0
Poly(ethylene oxide)	1-Propanol	10700	11450	343.2	393.2	3	6	4.3	3.0	8.2		3.0
Polystyrene	1-Propanol	20000		435.5	502.6	4	35	40.7	26.3			26.3
Polybutadiene	1-Propanol	93000	99000	339.2	369.2	4	3.8	17.4	17.8		32.7	17.8
Poly(methyl methacrylate)	Methanol	6107	6412	423	473	3	7.1	78.2	21.2	5.0		21.2
Polystyrene	Acetic acid	20000		435.5	502.6	4	76.2	47	18.1			18.1

Table S6: Comparison between the pre-trained GH-GNN model and the phenomenological models UNIFAC-ZM and Entropic-FV according to the mean absolute percentage error of Ω_{ij}^{∞} on the systems with association. All GH-GNN models here correspond to the ones tested for extrapolation. "G-" denotes the GH-GNN model trained on the corresponding data set. The last column corresponds to the reported performance as in the manuscript. The predictions from the phenomenological models were taken from the literature [2]. "#" denotes the number of points.

Solute	Solvent	MN	MW	T_1	T_2	#	UNIFAC-ZM	Entropic-FV	G-MN	G-MW	G-MN/MW	GH-GNN
Poly(ethylene oxide)	1-Butanol	2000	2180	348.2	398.2	3	39.1	15.1	19.1	10.5		19.1
Poly(ethylene oxide)	1-Propanol	10700	11450	343.2	393.2	3	6	4.3	6.8		16.4	6.8
Polystyrene	1-Propanol	20000		435.5	502.6	4	35	40.7	16.8			16.8
Polybutadiene	1-Propanol	93000	99000	339.2	369.2	4	3.8	17.4	41.8		11.1	41.8
Poly(methyl methacrylate)	Methanol	6107	6412	423	473	3	7.1	78.2	16.6	95.0	21.9	16.6
Polystyrene	Acetic acid	20000		435.5	502.6	4	76.2	47	32.6			32.6

References

[1] Wen Hao, H.S. Elbro, and P. Alessi. *DECHEMA Chemistry Data Series Vol. XIV. Polymer Solution Data Collection.* 1993.

[2] Georgia D Pappa, Epaminondas C Voutsas, and Dimitrios P Tassios. “Prediction of activity coefficients in polymer and copolymer solutions using simple activity coefficient models”. In: *Industrial & Engineering Chemistry Research* 38.12 (1999), pp. 4975–4984.

[3] Bernard Riedl and Robert E Prud’Homme. “Thermodynamic study of poly (vinyl chloride)/polyester blends by inverse-phase gas chromatography at 120° C”. In: *Journal of Polymer Science Part B: Polymer Physics* 24.11 (1986), pp. 2565–2582.
